# Sequential search asymmetry: Behavioral and psychophysiological evidence from a dual oddball task

**DOI:** 10.1371/journal.pone.0173237

**Published:** 2017-03-09

**Authors:** Elizabeth G. Blundon, Samuel P. Rumak, Lawrence M. Ward

**Affiliations:** 1 Department of Psychology, University of British Columbia, Vancouver, British Columbia, Canada; 2 Brain Research Centre, University of British Columbia, Vancouver, British Columbia, Canada; Harvard Medical School, UNITED STATES

## Abstract

We conducted five experiments in order to explore the generalizability of a new type of search asymmetry, which we have termed *sequential search asymmetry*, across sensory modalities, and to better understand its origin. In all five experiments rare oddballs occurred randomly within longer sequences of more frequent standards. Oddballs and standards all consisted of rapidly-presented runs of five pure tones (Experiments 1 and 5) or five colored annuli (Experiments 2 through 4) somewhat analogous to simultaneously-presented feature-present and feature-absent stimuli in typical visual search tasks. In easy tasks feature-present reaction times and P300 latencies were shorter than feature-absent ones, similar to findings in search tasks with simultaneously-presented stimuli. Moreover the P3a subcomponent of the P300 ERP was strongly apparent only in the feature-present condition. In more difficult tasks requiring focused attention, however, RT and P300 latency differences disappeared but the P300 amplitude difference was significant. Importantly in all five experiments *d*’ for feature-present targets was larger than that for feature-absent targets. These results imply that sequential search asymmetry arises from discriminability differences between feature-present and feature-absent targets. Response time and P300 latency differences can be attributed to the use of different attention strategies in search for feature-present and feature-absent targets, indexed by the presence of a dominant P3a subcomponent in the feature-present target-evoked P300s that is lacking in the P300s to the feature-absent targets.

## Introduction

What is the relationship between search through a static environment and search through a dynamic environment? Do we use similar mechanisms to look for features in a dynamic environment as in a static environment? In particular, does search through dynamic environments lead to asymmetries with respect to the presence or absence of particular features in the stimuli as it does in static environments? What do such asymmetries have to do with the P3a and P3b event-related potential (ERP) components? Moreover, the P3a is supposed to be related to the attention orienting response caused by a rare, unpredictable but very salient *distractor* stimulus. What happens when such a stimulus is not a distractor but instead signals the occurrence of a sought-after *target*? We address such questions in the context of a novel “search” paradigm utilizing longer sequences comprised of brief runs of auditory and visual stimuli. These stimuli are similar enough to previously used ones to invoke similar explanatory principles but different enough to shed new light on some old problems.

We had replicated, conceptually and for a different purpose, a study by Bekinschtein and colleagues [1]. That study used a simple oddball paradigm, but adapted to consist of two parts: the first part required that participants identify rare tone patterns among more frequent ones, while the second part required participants identify only simple changes in tone frequencies. This adaptation had the original intention of potentially assessing consciousness in participants who were unable to behaviorally respond to their environment, namely patients who were in an Unresponsive Wakeful State (UWS) or patients who were Minimally Conscious (MC). The first part of the task required conscious attention to perform, whereas the second part did not. The simple changes in tone frequencies generated a standard mismatch negativity (MMN) ERP response in the absence of completely intact consciousness, as it occurred in vegetative and minimally conscious patients. Changes in tone *patterns*, however, generated a P300 ERP response only in conscious controls and weakly in a few minimally conscious patients. These data suggest that detecting this kind of pattern change is not automatic and does require somewhat intact consciousness and sustained focal attention.

While performing our replication of the Bekinschtein et al. [1] protocol in typical conscious control participants, we noticed a possible double dissociation when participants were asked to identify pattern changes. The Bekinschtein et al. [1] protocol utilized two types of sequences of rare and frequent tone patterns. In one sequence the rare tone pattern contained a change in acoustic frequencies and the frequent pattern did not; in the other sequence the frequent tone pattern contained a change in acoustic frequencies but the rare pattern did not. Many of the participants in our replication reported informally during debriefing that they found it much easier to detect the rare pattern when it included an acoustic frequency change, than when it did not. The frequency-deviant tones seemed to pop out of a sequence of unchanging tones, while rare unchanging tone patterns seemed to be buried within a sequence of frequent, changing tone patterns, making them difficult to hear without attention being focused on each tone run.

These two different stimulus sequences thus seem to make very different demands upon attention. Detecting rare patterns that include a tone change would seem to require only diffuse voluntary (top-down) ongoing attention to the sequence of unchanging patterns, as the deviant tone in a rare changing pattern easily recruits bottom-up attention when it is encountered. In contrast, detection of the rare patterns that lack a tone change would seem to require sustained, voluntary (top-down) focused attention to each pattern in a sequence mostly comprised of patterns that possess a tone change, as there is no tone deviant to recruit bottom-up attention in the unchanging pattern [2].

**Fig 1:**
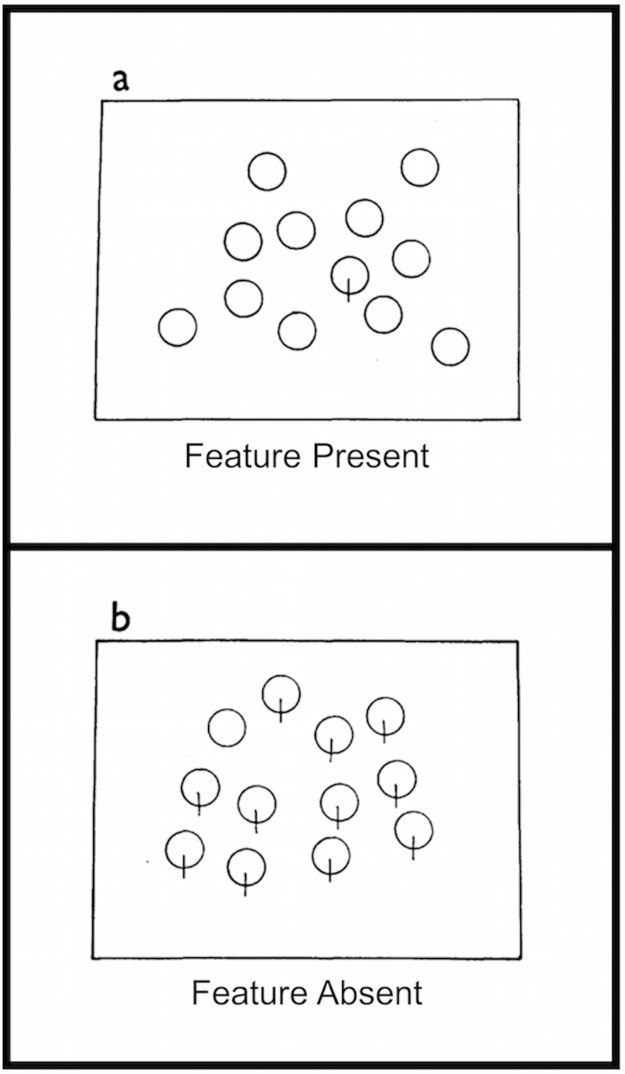
Example of search asymmetry stimulus. In the feature-present condition (a) the target is a symbol like the letter Q (circle with a line) while the distractors are O’s. In the feature-absent condition (b) the target is an O while the distractors are Q-like symbols Reprinted with permission from [4].

This speculation is reminiscent of a large body of work on *visual* search asymmetry phenomena, begun by Neisser [3] and popularized by Treisman and colleagues [4,5] as a technique to identify primitive features in the visual system. Treisman and Souther [4], in particular, found that certain visual features seem to be detected "preattentively," meaning that search for them seemed to be performed simultaneously and in parallel, and that they "popped out" when presented among items that lacked that feature. In this *feature present* condition the item with the extra feature popped out, and search time was consistently low regardless of number of distractors. For example, a symbol like the letter Q pops out amongst an array of Os (Fig 1). When the target was missing a feature that the distractors possessed (*feature absent* condition), however, search for a target with a missing feature was said to be serial and self-terminating, and search time varied with the number of distractors. Search time for an O amongst an array of Q-like symbols, for example, is longer and increases with the number of Qs. This kind of search asymmetry is consistent with Treisman’s feature integration theory [6], as items with a unique feature stand out in the relevant feature map and are quickly and easily detected, but items lacking that same feature presented in a context of items with multiple features take longer to detect because they do not stand out in any feature map.

The two rare auditory pattern detection tasks from our replication of the Bekinschtein et al. [1] study and the visual search tasks designed by Treisman and Souther [4] are similar in several ways (although different in others). First, in both tasks participants were asked to identify targets among a set of distractors, albeit the distractors in the Bekinschtein, et al. task are presented serially. Second, the auditory and visual stimuli that generated these sets contain some critical similarities. The auditory pattern sets consist of two almost identical auditory objects, each comprised of a run of five pure tones. The fifth tone of each object is either identical to the previous four (termed a *flat* run) or is different from them in frequency (a feature change, termed a *change* run). These auditory stimuli aren’t completely analogous to the visual stimuli, however, as what differs between the visual stimuli is the addition of a feature, not a feature change. Indeed, the auditory frequency of the tones in our stimuli is what Treisman and Souther [4] called a ‘substitutive feature,’ in which the absence of a feature in a stimulus implies the presence of another, for example color in visual stimuli. Nonetheless, acoustic frequency is mapped in the cortex to a tonotopic pitch map in which activation of different pitch regions corresponds nicely to activations of different regions in the shape maps in visual cortex [7]. Thus, finding the rare change run amongst the frequent flat runs is easy, because it contains a single different pitch feature that pops out in the pitch map; this is similar to finding the Q amongst the array of O’s. But finding the rare flat run amongst the frequent change runs is much more difficult because the change runs repeatedly and persistently activate two regions of the pitch map, and it is difficult to discriminate from them a stimulus that only activates a single one of those regions when both regions can still be somewhat active. This is like finding the O amongst an array of Q’s.

One critical difference between the paradigms is that distractors are not simultaneously present in the Bekinschtein et al. paradigm. We will deal with this difference in what follows. Another is that it is difficult to manipulate distractor set size in order to confirm a flat search slope for pop-out targets. Thus, other techniques must be used to identify different search strategies, as parallel processing of the entire array is ruled out by the serial nature of the stimulus presentation. Electrophysiological measures will help in this regard.

Luck and Hillyard [8] conceptually replicated Treisman and Souther’s original visual search task and found different P300 responses to the parallel (easy) and serial (difficult) visual search tasks [8]. Given the apparent differences in attention demands of the two auditory pattern search tasks described above, it seems likely that there should be different ERP responses to the two auditory pattern types, as in the two visual array types in the Luck and Hillyard [8] study. Thus, unlike Bekinschtein et al. [1] we need to analyze the P300 response for the two pattern types separately in order to uncover evidence of an ERP correlate of a type of search asymmetry in this paradigm. Moreover, we wondered whether this asymmetry exists for visual stimuli presented in the same way. Finally, we wondered whether the asymmetry would persist if the task were made so difficult that a diffuse attention strategy would not suffice to perform the task in the feature-present condition. If the same, focused attention, search strategy were required to detect both feature-present and feature absent targets, then any differences in search speed, accuracy, or the P300 responses to targets, could not be attributed to *differences* in search strategy. Rather some other feature of the stimuli and/or task must be responsible for those differences. One rather obvious possibility, given the earlier discussion of auditory and visual feature maps, would be that feature-present targets stand out more in the relevant feature maps in the brain. Hereafter, we sometimes refer to this possibility as a “perceptual” locus for the asymmetry, emphasizing that some aspect of perceptual processing, unrelated to any *difference* in attention strategy, is responsible for the observed differences in response to targets in the different conditions.

Of special interest here, is the study by Luck and Hillyard [8]. They found that the P300 ERP peak latencies and the behavioral reaction times to the feature-present target remained fairly constant regardless of set size, whereas P300 peak latencies and behavioral reaction times to the feature-absent target increased as a function of set size. What’s more, P300 amplitudes decreased as a function of set size for the feature-absent condition, whereas P300 amplitudes in the feature-present condition remained constant. Their behavioral reaction time results were consistent with those from Treisman and Souther’s original behavioral study, and together with the electrophysiological results Luck and Hillyard concluded that different strategies must be employed in the different conditions.

In addition to peak latencies and amplitudes differing, however, the *shapes* of the P300 responses in Luck and Hillyard’s [8] experiments were very different between feature-present and feature-absent conditions. The P300 ERP to the feature-present target was both larger and sharper than that to the feature-absent target. These different shapes suggest that the neurological processes underlying detection of these targets may be somewhat different. It has been argued that the P300 contains sub-components that are associated with distinct neurological processes [9,10]. One component, the P3a (or novelty P300), is most clearly revealed in association with detection of task irrelevant oddballs or “distractors,” which are rare, highly salient, unexpected stimuli that are not targets, and therefore typically do not incur a response from participants [11]. The P3a is also associated with the perception of any deviant or novel event [9]. The P3a is typically quite sharp and can occur as early as 200 ms after stimulus onset. It is strongest at centro-parietal electrode sites and can extend as far anterior as FZ. The P3a is believed to originate from fronto-parietal attention mechanisms that are involved in orienting attention to highly salient, unexpected stimuli [11]. A second component, the P3b, is broader and peaks later than the P3a, and is associated with task-relevant oddball or target identification. Its amplitude is maximal over parietal sites, but is quickly attenuated as it travels away from PZ. It is believed to originate from temporo-parietal networks which are thought to reflect memory and stimulus encoding mechanisms associated with context updating [10], among other processes. Though each of these sub-components has been investigated on its own, it is believed that “…every ‘P300’ is composed of the P3a and P3b subcomponents but the resulting ERP scalp topographies vary with the stimulus and task conditions that elicit them (caption of Figure 8 in [10], p. 2138)” (see also Figures 1, 5 and 8 in [10]). In other words, P300 shapes and latencies are highly sensitive to task demands because different tasks will require different demands on attention (P3a) and memory (P3b), which will generate P3a and P3b responses at different strengths. Given the shapes and scalp distributions of the P300 ERPs observed by Luck and Hillyard [8], it is likely that the P300 response to the feature-present target contained a stronger P3a subcomponent than the P300 response to the feature-absent target, which contained primarily the P3b subcomponent. We will return to this point when discussing the results of the present experiments.

Search asymmetries have been discovered in other sensory modalities as well. Several studies have confirmed similar asymmetries in haptic search [12–15], but these will not be reviewed here. Most relevant to the present paper is that Cusack and Carlyon [16] found that the auditory system was subject to a similar type of “search” asymmetry as the visual system. They created an auditory soundscape consisting of tones distributed in frequency and time (over 2 seconds). Their stimuli were comprised either of pure tones of various frequencies with or without a frequency-modulated (warble-tone) target, or of warble-tones with or without a pure-tone target. Participants were more accurate (larger *d*’) in detecting the warble-tone target among the pure-tone distractors than in detecting the pure-tone target among the warble-tone distractors. In the context of a number of other variations designed to rule out some peripheral auditory processing mechanisms as the source of this asymmetry, they concluded that the use of different search strategies was the best explanation for the auditory search asymmetry that they had observed.

The mismatch negativity (MMN) has been used to investigate how the auditory system responds to deviations in tone patterns in long sequences [17–19]. A few of these have used the MMN directly to investigate whether the origins of search asymmetry such as that found by Cusak and Carlyon lie in early perceptual (automatic, or at least not arising from differences in attention strategies) or attention strategy mechanisms in audition [20,21] and vision [22]. Although these studies provide evidence that feature-present targets trigger larger MMNs than do feature-absent targets, and can do so automatically (i.e., when attention is focused elsewhere prior to stimulus presentation), they do not account for behavioral differences between the conditions, suggesting that a different or at least additional mechanism must be associated with the asymmetrical behavioral responses. It is this additional mechanism with which we are concerned. Given that the MMN appears not to predict the behavioral differences observed, we were particularly interested in whether the difference in the P300 response to feature-present and feature-absent stimuli first found by Luck and Hillyard [8] would do so.

Five experiments were conducted to address the above-described issues. As mentioned in the introduction, the first was a conceptual replication of the Bekinschtein et al. [1] auditory paradigm, where the sequential nature of the search task was made explicit and separate analyses were conducted for the P300 responses to feature-present and feature-absent conditions. The second and third were modified versions of the auditory experiment but using visual stimuli instead of auditory stimuli. These experiments were designed to discover whether sequential “search” asymmetry is a property of the auditory system or if it generalizes to the visual system as well. The fourth and fifth experiments served as a combined first step into investigating the mechanism giving rise to the asymmetry: a difference in attention strategy or a difference in stimulus discriminability, or perhaps elements of both.

To date, attention-orienting models involving the P3a have only been associated with attention orienting *away* from a participant’s current task, because the P3a is typically studied as a response to highly salient and unpredictable *task-irrelevant* oddballs. The P3a contribution to P300 responses made to rare salient and unpredictable *task-relevant* oddballs, and subsequent behavioral responses, has therefore been largely ignored. Exploring the relationship between P3a/P3b and behavioral responses to task-relevant oddballs may provide a deeper understanding of when and how the proposed attention-orienting mechanisms are used during search tasks, and in particular how they may be associated with search asymmetry behavior.

## Experiment 1: Auditory sequential search

As mentioned in the introduction, Experiment 1 was a conceptual replication of the study by Bekinschtein and colleagues [1]. The Bekinschtein et al. [1] protocol was ideal to investigate auditory search asymmetry because it consisted of two different rare auditory pattern detection tasks that are in some ways similar to the visual search tasks designed by Treisman and Souther [4] and also to the previous auditory search studies [16,20,21]. We modified this paradigm so that flat runs consisted of five repetitions of the same pure tone and change runs consisted of four of the same tone followed by a different-frequency fifth tone. Thus, finding a rare change run among common flat runs should be easy because it contains a different pitch feature that should pop out in the pitch map; this is similar to finding a Q among an array of O’s. But finding a rare flat run among common change runs should be much more difficult because the change runs repeatedly and persistently would activate two regions of the pitch map and it is difficult to discriminate from them a stimulus that activates only one of those regions when both regions could still be somewhat active. This is like finding an O among an array of Q’s. This is similar to the explanation that Treisman and Souther [4] gave for their visual search asymmetry results.

Bekinschtein et al. [1] established that the P300 waveform is generated in response to successful identification of the global pattern change. They did not collect behavioral (reaction time or accuracy) data, however, nor did they separate their P300 results into feature-present and feature-absent conditions. Instead they collapsed the data across those conditions. To illustrate this, the visually analogous situation would be to treat search for a Q among O’s as equivalent to search for an O among Q’s, and to report a single P300 response to identification of all targets regardless of whether they were a Q or an O. As was mentioned in the introduction, Luck and Hillyard [8] conceptually replicated Treisman and Souther’s original visual search task and found *different* P300 responses to the easy (for example search for a Q among O’s) and difficult (search for an O among Q’s) visual search tasks. Given the apparently different demands of the two auditory pattern search tasks described above it seems likely that there should be different ERP responses to the two auditory pattern types (changing patterns embedded in unchanging ones and unchanging patterns embedded in changing ones) as in the two visual array types in the Luck and Hillyard [8] study. Thus, unlike Bekinschtein et al. [1], we analyzed the reaction times and P300 responses for the two pattern types separately to uncover evidence of search asymmetry in the auditory domain, and in a task where the sequential nature of the “search” was made explicit.

### Methods

#### Participants

Data were collected initially from 20 participants who were recruited via the UBC Psychology Department’s online experimental participant recruitment site and via a poster on the Department’s participant recruitment bulletin board. From 15–25 participants has been shown in previous studies in our lab and others to yield reliable EEG data for either ERP or connectivity analyses given the numbers of stimulus trials in the study design. Data collection was stopped after the indicated number of participants had been included. Data from three participants were excluded because of excessive noise in their EEG. Thus the analysis to be described is based on 17 participants (10 female, age 18 to 30 years, mean age 23.1 years). All aspects of the experimental protocol, including the recruitment and consent procedures, were approved by the University of British Columbia Behavioural Research Ethics Board in accordance with the provisions of the World Medical Association Declaration of Helsinki. Participants gave written informed consent by reading and signing the approved consent document, and were offered monetary compensation ($10/hr) for their participation. Participants were all right-handed and reported no hearing or neurological difficulties.

#### Stimuli

**Fig 2:**
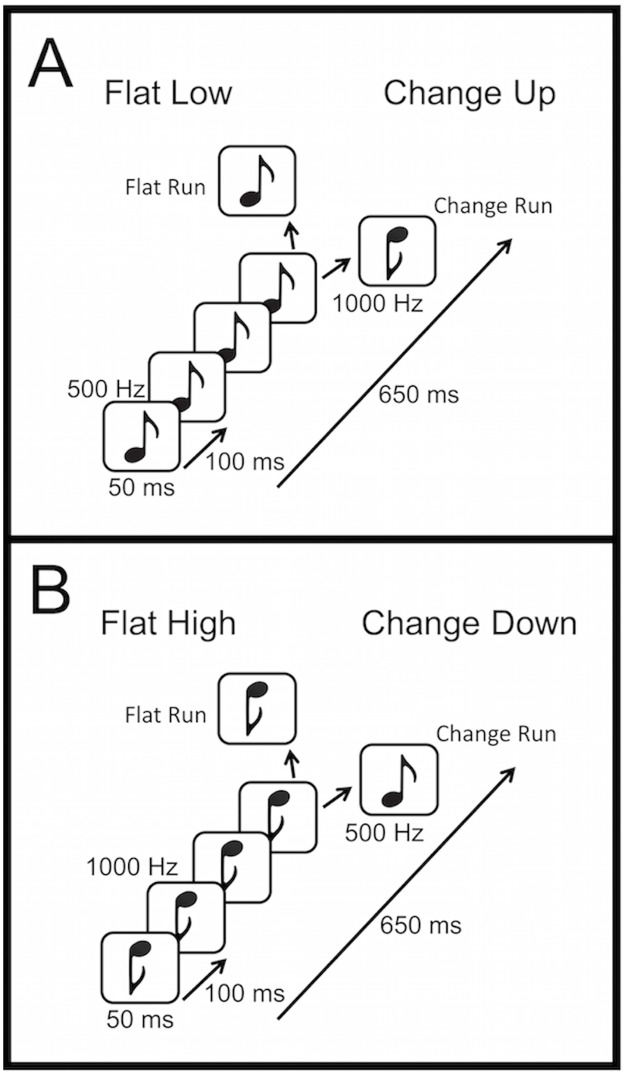
Experiment 1 pure-tone stimuli. Stem-down high notes are 1000 Hz and stem-up low notes are 500 Hz. Flat runs consisted of five pure tones of the same frequency; change runs consisted of four pure tones of the same frequency followed by a fifth tone of a different frequency.

We generated 50-ms duration pure tones with 7.5 ms onset and offset ramps, using the ascending first half of a Hann window for the onset and the descending second half of the Hann for the offset, using a custom MATLAB (MathWorks, Natick USA) script. The tones were administered binaurally at 70 dB through insert earphones (EAR 3A) in a sound-attenuating chamber. Stimuli were presented and responses registered using Presentation software (Neurobehavioral Systems Berkeley CA USA). Tone runs were generated using Audacity (Sourceforge). Auditory stimuli consisted of two types of five-tone runs called *flat runs* and *change runs* (see Fig 2). Flat runs consisted of five pure tones of the same frequency while change runs consisted of four pure tones of the same frequency followed by a fifth tone of a different frequency. All runs consisted of a combination of 500-Hz and 1000-Hz tones which generated two versions of each type of run: one in which 1000-Hz tones comprised the first four tones in a change run and all the tones in the flat run (*change-down* and *flat-high* see Fig 2B), and the same for the 500-Hz tones (*change-up* and *flat-low* see Fig 2A). Successive 50-ms duration tones in a run were separated by 100 ms of silence. Thus each run lasted 650 ms from the onset of the first tone to the offset of the last tone. Intervals between the offset of the final tone of a given run and the onset of the first tone of the next run varied randomly from 700 ms to 1000 ms.

#### Procedure

**Fig 3:**
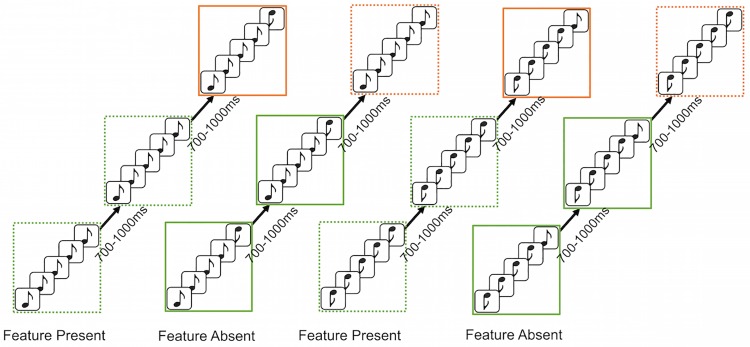
Experiment 1 sequence design. Participants each heard four sequences consisting of two feature-present sequences and two feature-absent sequences. Stem-down high notes are 1000 Hz and stem-up low notes are 500 Hz. Rare runs are targets to be detected in the longer sequence of common runs. Green borders are around common runs; orange borders are around rare runs. Dashed lines are around flat runs; solid lines are around change runs.

Once participants were comfortably seated in the sound-attenuating chamber they were familiarized with the stimuli and protocol. Each participant heard four extended oddball sequences of tone runs (about 35 mins per sequence) in randomized order per subject (see Fig 3). Each sequence began with 30 instances of the common run. From then on the rare run was presented on a random 20% of occasions among 80% common runs. In each sequence rare runs were heard between 18 and 30 times. There were always at least 2 common runs before and after each rare run. The four sequences of runs consisted of the following: (1) common flat-low, rare change-up; (2) common change-up, rare flat-low; (3) common flat-high, rare change-down; (4) common change-down, rare flat-high. Participants were instructed to click a mouse every time they heard a pattern oddball (rare run) and their response times were recorded. It should be noted that it was made clear to participants that they were only to respond when they heard a change in the global pattern, i.e. a rare run, *not* every time they heard a tone that differed from the previous tone.

#### EEG recording and ERP extraction

EEG signals were digitized at 500 Hz (National Instruments Inc., Vaudreuil-Dorion QC Canada) from a 60-channel electrode cap (Electrocap Inc., Eaton OH USA, International 10–10 placement) referenced to the right mastoid. Before digitization EEG signals were amplified and analog bandpass filtered from 0.1 Hz to 100 Hz (SA Instrumentation, San Diego CA USA). Eye movements were recorded with four periocular electrodes. All electrode impedances were kept below 10 kΩ (input impedance of the amplifier was > 2 gΩ)

EEG data were analyzed using EEGLAB software [23]. First they were down-sampled to 250 Hz and re-referenced to average reference. Artifacts were rejected using independent component analysis (runica algorithm EEGLAB; [24]). The remaining ERP analyses were conducted using ERPLAB [25], ERP analysis software that runs in EEGLAB. The continuous EEG record for each individual subject was then epoched for ERP analysis from -1000 to +2000 ms relative to the onset of the first tone of each run, low-pass filtered at 30 Hz using a FIR filter, and baseline corrected (-200 to 0 msec re onset of fifth tone in each run). Shortened segments of these epochs were selected for display purposes, particularly when only the final tone in a run was of interest. All epochs around rare runs and the epochs around the common runs that appeared immediately before the rare runs were extracted. This ensured equal numbers of common- and rare-run-epochs for the latter analyses.

#### ERP component measurement

P300s were characterized as difference waves between a P300 response to the final tone of a run when that run was the target run compared to when that run was the standard non-target run. For example, we used a change run when that run was the rare target run and that same run when it was the frequent standard run to characterize a feature-present P300. This allowed a determination of the effect of the longer-sequence role alone on the P300, independent of the physical makeup of the run. Latencies were determined by measuring the time difference between the onset of the final tone in a run and the moment of the maximum amplitude of the average difference wave between 200–1000 ms after final tone onset, for each participant separately. This range represents rough estimates of the beginning and end of the entire P300 ERP morphology, taken from the grand average difference wave (average ERP from all participants) of each condition from the zero crossing where the potential begins to ascend from zero to the second zero crossing where the potential descends to zero. Amplitudes were determined by taking the average amplitude of the grand average difference wave (as above) over an interval 20 ms to either side of the maximum, for each participant separately. We did not do adaptive filtering, either here or in the following experiments, to compensate for latency jitter [26,27]. Previous investigations of the P300 in visual search asymmetry [8] found that adaptive filtering didn’t give useful results, and a previous investigation of the usual 3-stimulus P3a/P3b paradigm found that compensating for jitter did not materially affect measurement of those components [28]. The possibility that latency jitter differences between conditions could be responsible for any amplitude differences found in what follows, therefore, must remain a topic for future research.

### Results

In what follows, to be consistent with previous terminology, here and throughout the paper we call the two sequences in which the rare runs were the flat runs (flat-low and flat-high) the ‘feature-absent’ condition, and those in which the rare runs were the change runs (change-up and change-down) the ‘feature-present’ condition even though “feature-change absent” and “feature-change present” is what is meant. Mean response times and ERP latencies and amplitudes were compared with MANOVA for repeated measures with *η*^2^ as effect size for the overall analysis and partial *η*^2^ (*η*^2^_*p*_) for the univariate *F*-tests for planned comparisons (see Table 1).

**Table 1:** Experiment 1 RT and electrophysiological results.

Target Run	RT (ms)	P3 Lat (ms)	P3 Amp (μV)
**Change-Up (CU)**	386	348	8.8
**Flat-Low (FL)**	542	499	7.2
**Difference**	156	151	1.6
***p***	0.0003*	0.002*	0.15
**u*F(1*,*16) (η*^*2*^_*p*_*)***	21.45(0.57)	13.59(0.46)	N/A(N/A)
**Change-Down (CD)**	386	381	8.2
**Flat-High (FH)**	556	500	7.4
**Difference**	170	119	0.8
***P***	0.0004*	0.02*	0.37
**u*F(1*,*16) (η*^*2*^_*p*_*)***	19.40(0.55)	6.42(0.29)	N/A(N/A)

Results are based on the average of all individual differences between conditions. Significant differences are indicated with an asterisk (*). Effect size (*η*^*2*^_*p*_) estimates and unvariate Fs (u*F*) are only included for significant differences.

#### Behavioral results

Table 1 summarizes the response time results of the experiment. The response times to correctly detect the rare target run (hits) were calculated from the onset of the fifth tone in the run separately for the two stimulus types (flat-low and flat-high) comprising the feature-absent condition, and the two stimulus types (change-up and change-down) comprising the feature-present condition. In each case response latency was significantly longer in the feature-absent condition (targets flat-low or flat-high) than in the corresponding feature-present condition (targets change-up or change-down). Correct responses were defined as a response to a rare target run that occurred before the onset of the final tone of the following non-target run. This time window varied between 1300 ms and 1900 ms after the onset of the final tone in a target run. Because some subjects had zero false alarms we undertook a pooled (pooled across subjects and across all feature-present targets and across all feature-absent targets) signal detection theory analysis [29]. In this analysis a single hit proportion and a single false alarm proportion are computed for each condition and the resulting *d*’s are compared using a variance estimate based on the number of overall signal present and signal absent trials in this yes-no-type design. The results of this analysis for Experiment 1 and those for all subsequent experiments are given in Table 2. It is clear that *d*’ in Experiment 1 differs between the two conditions although both *d*’s are large, indicating excellent discriminability overall. Nonetheless these results indicate that discriminability of feature-present targets is significantly greater than that of feature-absent targets. This result is consistent with the response latency results and indicates that there was no large speed-accuracy tradeoff.

**Table 2:** Results of pooled *d*’ analysis.

Experiment	Condition	*d*’	*d*’ difference	*Z*-score	*p* <
**E1 Auditory**	FP	5.240	1.195	3.98	0.0001
	FA	4.045			
**E2 Color**	FP	5.180	0.84	2.8	0.003
	FA	4.340			
**E3 BW**	FP	5.665	1.555	5.18	0.0001
	FA	4.110			
**E4 Grey**	FP	3.135	0.595	5.43	0.0001
	FA	2.540			
**E5 Rov Aud**	FP	3.520	0.72	5.37	0.0001
	FA	2.800			

#### P300 ERP results

**Fig 4:**
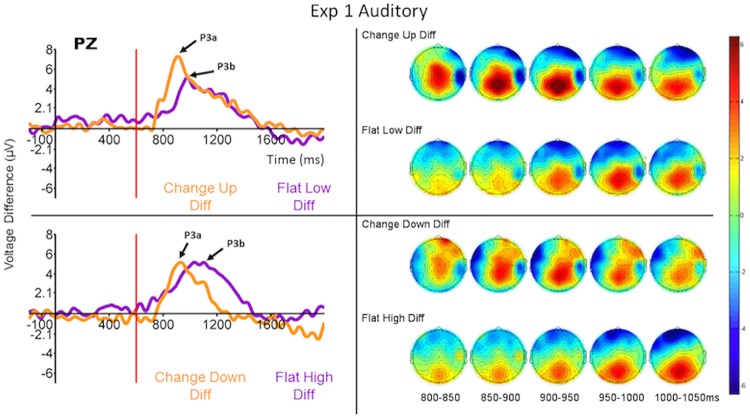
Grand average (across all subjects) ERP difference waves at Pz (P300—left) and topomaps (right) to the four run types when they were rare or common. Each panel compares the difference waves for feature-present and feature-absent conditions for run types in which the first four tones in the run were identical. (Top) Change-up (feature-present) compared to flat-low (feature-absent). (Bottom) Change-down (feature-present) compared to flat-high (feature-absent). Time 600 ms refers to the onset of the last tone of the run. The ERPs from which the difference waves were derived are displayed for these and other selected electrode sites in S1 Fig.

We computed difference waves from the ERPs to the final tone (onset at 600 ms relative to the onset of the first tone) of each run type when that run type was rare and when it was common. These difference waves reveal the ERP effect of detecting a feature-present target or feature-absent target in the absence of any physical differences between the stimuli. The difference waves comparing the ERPs to the identical run type when it was rare and when it was common are displayed in Fig 4 (left side) for the Pz electrode and the ERPs themselves are displayed in S1 Fig for a selection of relevant electrodes (note: the ERPs in supplementary figures are paired by condition not by run type). All grand average ERP figures were filtered at 15 Hz using a FIR filter for display purposes only.

The overall effect of the feature-present vs feature-absent factor (i.e., change-down and change-up vs flat-low and flat-high runs) for P300 latency was significant in the repeated measures MANOVA (*F*_1,16_ = 17.44, *p* = 0.001, *η*^2^ = 0.27). No other effects were significant. Table 1 displays the results of the univariate *F*-tests for each of the (planned) comparisons described above.

Fig 4 demonstrates that there was a clear P300-like response peaking between about 300 ms and 500 ms post onset of the fifth (last) tone in each run (900 ms and 1100 ms in the figure) to all runs when they were rare. There was no such response when those same runs were common—see S1 Fig. The difference waves, and consequently the latencies of the peaks of both the P300 responses and the difference waves, differed substantially, however, between the flat (feature-absent) and change (feature-present) runs. The feature-present target P300 ERP was much sharper and peaked significantly earlier than the corresponding feature-absent target P300 ERP (see also Table 1). On the other hand the amplitudes of the difference waves were not significantly different. We also analyzed the differences between the areas under the P300 curves (numerical integration of the curves) and the results were similar to those just detailed and so are not reported here.

In addition to the difference in peak latency just described, the P300 response differed in scalp topography between feature-present and feature-absent conditions (see right side of Fig 4 and S1 and S6 Figs). For both types of feature-present targets (change-up and change-down) the P300 had the earliest and sharpest peak at medial frontal sites, and its amplitude declined only slowly from Pz to Fz electrodes. For both types of feature-absent targets (flat-low and flat-high), however, the amplitude of the broader and later difference wave was largest at Pz and attenuated more quickly toward more frontal sites. A MANOVA for repeated measures (collapsed across change-up and change-down for feature-present and across flat low and flat high for feature-absent) showed that the interaction between condition (feature-present vs feature-absent) and electrode (Fz, FCz, Cz, CPz, and Pz) was significant (*F*_4,64_ = 2.65, *p* = 0.04, *η*^2^ = 0.02). The planned contrast between conditions at electrodes Fz and FCz also was significant (*F*_1,16_ = 14.02, *p* = 0.002, *η*_*p*_^2^ = 0.53). This result is consistent with the presence of a larger P3a subcomponent in the P300 response in the feature-present condition that was lacking in the feature-absent condition. There were also significant main effects of condition as described above (*F*_1,16_ = 12.31, *p* = 0.003, *η*^2^ = 0.08) and electrode (*F*_4,64_ = 8.56, *p* = 0.00001, *η*^2^ = 0.10).

### Discussion

The results of Experiment 1 constitute additional evidence for search asymmetry in the auditory domain. Our response time results were similar to those of the visual search task designed by Treisman and Souther [4]; targets that possessed a feature-difference that distractors lacked (feature-present condition) were identified faster than were targets that lacked a feature-difference that distractors possessed (feature-absent condition). Our P300 ERP results also were highly similar to those of Luck and Hillyard [8] for simultaneously-presented visual stimuli.

By subtracting the response when each stimulus was common from the response when the same stimulus was rare, we can see the P300 response to the target category in the absence of any activity generated by task-irrelevant feature changes inherent in the stimuli. S1 Fig shows that there was virtually no P300 response to the common runs in both feature-present and feature-absent conditions, regardless of whether they were flat or change runs. Therefore the P300 difference waves (Fig 4) overlap nearly completely with the P300 responses to the rare stimuli (S1 Fig). This suggests that the P300 responses to the targets exclusively reflected processing of the pattern change. The differences between the difference waves for feature-present and feature-absent conditions, and the relative contributions of the P3a/P3b subcomponents to each, will be discussed further in the General Discussion in the context of all five experiments.

Treisman and Souther [4] and Luck and Hillyard [8] interpreted the differences in response times and P300 latencies between the conditions as indicative of participants using different search strategies for each of the conditions: pre-attentive parallel search in the visual feature-present condition and serial self-terminating search in the visual feature-absent condition. Because auditory stimuli in the present experiment were presented in series rather than simultaneously, however, there was no opportunity for our participants to use a parallel search strategy, nor could their search be self-terminating as participants were not free to search among all distractors at their leisure. Strictly speaking then the classic interpretation of the mechanisms that underlie the observed search behaviors do not apply in the sequential auditory search case. As mentioned in the Introduction, it is possible the asymmetry could simply be an artifact of the way in which the auditory system perceives auditory objects. It is possible, however, that the behavioral and electrophysiological similarities between these tasks reflect a difference in attention distribution strategies that can be applied to search through both space and time and in all sensory domains. Perhaps in feature-present conditions (and their analogs) a diffuse attention strategy can be applied where, because participants are looking (or listening) for a specific but rarely occurring salient feature, they need only attend superficially to each stimulus. And perhaps in feature-absent conditions a more focused attention strategy is applied where participants attend to every individual stimulus (or stimulus subset in a fixation) in order to integrate all features for identification. It is possible that these strategies manifest in what appear to be parallel or serial self-terminating search only when perceptual objects are presented simultaneously, or rather when the task requires participants to search through space rather than through time.

A plausible analogous strategy in our experiment is that auditory attention was distributed temporally, particularly timed to the final tone of each run, as it is presented in a predictable rhythm [30,31]. This rhythmic cue could facilitate deployment of attention to each tone in a run. This strategy could be crucial to identifying the feature-absent target, as deployment of focused attention at the correct temporal position of every run is required to identify whether a run is common or rare. Attention does not need to be voluntarily deployed to the end of each run in the feature-present condition, however, as the presence of the change tone, regardless of in which temporal position it appears, is itself sufficient to identify the target. Thus, in identification of a feature-present auditory target, attention can be deployed diffusely in time as it can be deployed diffusely in space in search for a feature-present visual target.

The following four studies were performed a) to confirm that the similarities between visual and sequential auditory search behavior discovered in Experiment 1 are generalizable to sequential search tasks regardless of stimulus modality (Experiments 2 and 3), and b) to investigate whether the effect in our sequential paradigm arises from differences in target-distractor discriminability between feature-present and feature-absent conditions, or rather if it only arises upon the deployment of different attention strategies to deal with different stimulus contexts (Experiments 4 and 5). Experiments 2, 3, and 4 are presented together as their stimuli and study designs are almost identical and their data were taken from the same group of participants. The data from these experiments, however, were analyzed separately, for reasons that will become clear. Experiment 5 is presented separately as it not only uses a very different design but also a different group of participants.

## Experiments 2, 3, and 4: Visual sequential search

For our visual analog of the auditory runs of Experiment 1, we created sequences of common and rare flat and change runs of visual stimuli that required serial search just as did the auditory runs. Experiments 2 and 3 were designed to confirm that sequential visual search yields comparable results to sequential auditory search showing that the sequential search results aren’t unique to the auditory system. Experiment 2 used color differences (yellow and blue) whereas Experiment 3 used contrast differences (black and white) to explore sequential search asymmetry. Both color and contrast are *substitutive features* [4] where the absence of one implies another. Experiments 2 and 3 were performed to make sure that any asymmetry found was not unique to a specific visual feature, but could generalize at least to all substitutive features. Experiment 4 was a first attempt to test attention orienting strategies described by Wright and Ward [2]. The intention of Experiment 4 was to force participants to voluntarily focus their attention on both common and rare runs, regardless of condition, by reducing the salience of the feature difference that defined each type of run. As in Experiments 2 and 3, the flat runs and change runs consisted of colored rings; however, in Experiment 4 the colors were two very similar but distinguishable shades of grey. Making the shades of grey very similar resulted in the flat and change runs being difficult to discriminate from each other. If detecting the feature-present targets only required diffuse ongoing attention then making the targets nearly indistinguishable from the distractors would force participants to focus their attention on every stimulus in the feature-present conditions. If detecting the feature-absent condition targets already required focused attention, then making those targets nearly indistinguishable from the distractors shouldn’t change participants’ strategy in that condition. By forcing participants to apply the same attention strategy to both conditions equally, the asymmetry operationally defined by a difference in reaction time, accuracy, and P300 latency and amplitude measures should be eliminated if the effect arises from the application of different attention strategies. If the asymmetry persists then differences in perceptual analysis of the two target types are likely responsible.

### Methods

#### Participants

Data were collected from 19 participants who all participated in Experiments 2, 3, and 4. Participants were recruited via the UBC Psychology Department’s online experiment participant recruitment site and via a poster on the Department’s participant recruitment bulletin board. From 15–25 participants has been shown in previous studies in our lab and others to yield reliable EEG data for either ERP or connectivity analyses given the numbers of stimulus trials in the study design. Data collection was stopped after the indicated number of participants had been included. Data from 3 participants were excluded due to excessive EEG noise or stimulus presentation error. The analysis is based on 16 participants (10 female, age 18 to 29 years, mean age 21.7 years). All aspects of the experimental protocol, including the recruitment and consent procedures, were approved by the University of British Columbia Behavioural Research Ethics Board in accordance with the provisions of the World Medical Association Declaration of Helsinki. Participants gave written informed consent by reading and signing the approved consent document, and were offered monetary compensation ($10/hr) for their participation. Participants were all right handed and reported no vision or neurological difficulties.

#### Stimuli

Visual stimuli were generated and presented using Presentation software (Neurobehavioral Systems Berkeley CA USA), and they appeared on an 18.5-in CRT monitor running at 77 Hz placed at a viewing distance of approximately 60 cm from the participant. Stimuli in each experiment consisted of two types of five ring (annulus) runs, flat runs and change runs. Flat runs consisted of five identical rings whereas change runs consisted of four identical rings followed by a fifth ring that differed by some feature. The fifth ring of the change runs differed in color in Experiment 2, in contrast in Experiment 3, and grey-scale value in Experiment 4. Runs consisted of a combination of 2 dimensions of the same feature: yellow (RGB = 255,255,0; luminance = 229.6 candela/m^2^) and blue (0,0,255; 219.3) in Experiment 2, black (0,0,0; 3.4) and white (255,255,255; 380.3) in Experiment 3, and two different shades of grey in Experiment 4, where the lighter shade was always (101,101,101; 58.3). The darker shade varied between (65,65,65; 41.1) and (51,51,51; 37.7) to accommodate each participant’s ability to discriminate between the flat and change runs at these low contrast levels. One participant required a larger difference: light (111,111,111; 68.5) and dark (35,35,35; 34.3).

**Fig 5:**
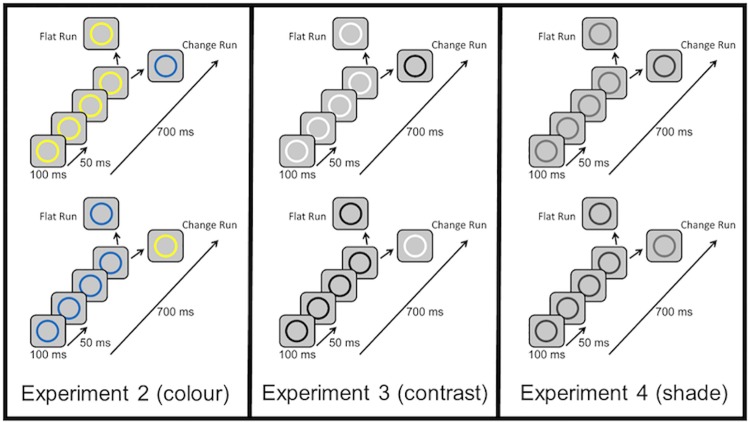
Annulus stimuli. All ring colors in the figure are approximations of ring colors presented to participants.

These combinations generated two versions of each type of run (see Fig 5). Rings were presented against an intermediate grey background; RGB values of the background were (200,200,200) for Experiments 2 and 4 and (128,128,128) for Experiment 3. Background shades were not consistent across all experiments because we tried to choose a background shade that would be in roughly equal contrast against each colored annulus. Unfortunately a single background color could not achieve that for all six colors. Each ring was presented for 100 ms and rings were separated by 50 ms of blank screen. Each run lasted 700 ms from the initial presentation of the first ring to the end of the last ring. Intervals between each run varied randomly from 700 ms to 1000 ms.

#### Procedure

The design used for each experiment was identical to that of Experiment 1 (see Fig 3 for a schematic of the design). The protocol used in Experiments 2, 3, and 4 was also very similar to that described in Experiment 1.

Once participants were comfortably seated approximately 60 cm from the monitor in the sound-attenuating chamber, they were familiarized with the stimuli and protocol. Next they participated in a short calibration for Experiment 4. During the calibration, 16 runs (2 of each type) were presented in the same random order and participants were asked to verbally identify each run by saying “flat” or “change.” The shades of grey were adjusted as necessary so that each participant could identify every run with near perfect (no less than 94%, 15/16 correct) accuracy. The calibration was repeated until each participant reached this accuracy threshold. This calibration was only performed for Experiment 4 because during a behavioral-only pilot run of this protocol participants reported that discriminating the stimuli in Experiments 2 and 3 was very easy without adjustment, whereas discriminating the stimuli in Experiment 4 was overall much more difficult and the difficulty varied across participants.

Each subject participated in all three experiments, and the order of their presentation was determined using a Latin square design. Each experiment consisted of 4 oddball sequences (about 4 mins each) presented in randomized order. Each sequence consisted of two run types: one common and one rare. Sequences began with 30 instances of the common run. Thereafter rare runs were presented randomly among common runs. Rare runs were presented on 20% of occasions and common runs were presented on 80% of occasions, so in each sequence participants saw between 21 and 29 rare runs (except that Experiment 3 one participant saw 16 rare runs in one sequenced and in Experiment 4 a different participant saw 39 rare runs in one sequence). At least two common runs occurred before and after each rare run. Participants were instructed to click a mouse every time they saw a rare run and their reaction times were recorded. It should be noted that it was made clear to participants that they were only to respond when they saw a rare run, not every time they saw a ring that differed from the previous ring.

#### EEG recording and ERP extraction

EEG recording and ERP extraction were nearly identical to that of Experiment 1 save for the latency windows from which the peak amplitudes were determined. The latency window for Experiments 2 and 3 was 200 to 700 ms and for Experiment 4 it was 350 to 750 ms after the onset of the last ring. As in Experiment 1 these ranges represent rough estimates of the beginning and end of the entire P300 ERP, taken from the grand average difference wave (average ERP from all participants) of each condition for each experiment.

### Results

Data analyses and terminology are identical to those used for Experiment 1.

#### Behavioral results

The response times to correctly detect the rare target run (hits) were calculated from the onset of the fifth ring in the run separately for feature-absent and feature-present conditions for all three experiments (6 of each in total). Tables 3 and 4 summarize the response time results of Experiments 2 and 3 respectively. In general Experiments 2 and 3 yielded similar results to those of the auditory Experiment 1. In Experiments 2 and 3 the reaction time to identify the feature-absent target was significantly longer than to identify the corresponding feature-present target. Corresponding conditions were those that shared stimuli (for example flat yellow) but whereas in one condition that stimulus was common (feature-present) in the other condition it was rare (feature-absent) and vice versa for the change yellow to blue. Correct responses were defined as a response to a rare target run that occurred before the onset of the final ring of the following non-target run. This time window varied between 1400 ms and 2000 ms after the onset of the final ring in a target run. Again because some subjects had zero false alarms a signal detection analysis of the pooled data (pooled across participants and also across specific stimuli) was undertaken. Table 2 shows that *d*’ was greater for the feature-present than for the feature-absent conditions for both Experiments 2 and 3, again consistent with the response latency results and ruling out a large speed-accuracy tradeoff.

**Table 3:** Experiment 2 RT and electrophysiological results.

Target Run	RT (ms)	P3 Lat (ms)	P3 Amp (μV)
**Change Yellow to Blue (Y2B)**	389	404	8.1
**Flat Yellow (Y)**	488	445	5.4
**Difference**	99	40	2.7
***p***	0.0000001*	0.26	0.004*
**u*F(1*,*15) (η*^*2*^_*p*_*)***	92.15(0.86)	N/A(N/A)	11.35(0.43)
**Change Blue to Yellow (B2Y)**	377	417	7.6
**Flat Blue (Blu)**	500	491	5,4
**Difference**	123	74	2.2
***p***	0.000001*	0.014*	0.06
**u*F(1*,*15) (η*^*2*^_*p*_*)***	66.44(0.82)	7.62(0.34)	4.09(0.21)

Results are based on the average of all individual differences between conditions. Significant differences are indicated with an asterisk (*). Effect size *(η*^2^_*p*_*)* estimates and univariate *F*s *(*u*F)* are only included for significant differences.

**Table 4:** Experiment 3 RT and electrophysiological results.

Target Run	RT (ms)	P3 Lat (ms)	P3 Amp (μV)
**Change White to Black (W2B)**	474	432	9.9
**Flat White (W)**	521	479	6.8
**Difference**	48	48	3.1
***p***	0.006*	0.16	0.01*
**u*F(1*,*15) (η*^*2*^_*p*_*)***	10.26(0.41)	N/A(N/A)	8.09(0.35)
**Change Black to White (B2W)**	382	395	9.5
**Flat Black (Bla)**	474	486	5.1
**Difference**	92	91	4.4
***p***	0.000002*	0.02*	0.004*
**u*F(1*,*15) (η*^*2*^_*p*_*)***	56.40(0.78)	6.57(0.30)	11.81(0.44)

Results are based on the average of all individual differences between conditions. Significant differences are indicated with an asterisk (*). Effect size *(η*^2^_*p*_*)* estimates and univariate *F*s *(*u*F)* are only included for significant differences.

In Experiment 4 the response latencies to the feature-present and feature-absent targets did not differ significantly (Table 5). False alarms, though larger than in Experiments 2 and 3, were still very infrequent: less than 3%. The pooled signal detection theory analysis, however, indicated that as in Experiments 1–3 *d*’ was significantly greater for feature-present targets than for feature—absent targets (Table 2).

**Table 5:** Experiment 4 RT and electrophysiological results.

Target Run	RT (ms)	P3 Lat (ms)	P3 Amp** (μV)
**Change Light to Dark (L2D)**	519	525	5.7
**Flat Light (L)**	549	545	3.9
**Difference**	30	20	1.8
***p***	0.25	0.63	0.012*
**u*F(1*,*15) (η*^*2*^_*p*_*)***	N/A(N/A)	N/A(N/A)	8.21(0.35)
**Change Dark to Light (D2L)**	540	528	7.2
**Flat Dark (D)**	561	537	6.4
**Difference**	21	9	0.8
***p***	0.57	0.80	0.23
**u*F(1*,*15) (η*^*2*^_*p*_*)***	N/A(N/A)	N/A(N/A)	N/A(N/A)

Results are based on the average of all individual differences between conditions Effect size *(η*^2^_*p*_*)* estimates and univariate *F*s *(*u*F)* are only included for significant differences. Significant differences are indicated with an asterisk (*). Multivariate P3 amplitude test was significant for feature-present versus feature-absent (**).

#### P300 ERP results at Pz

**Fig 6:**
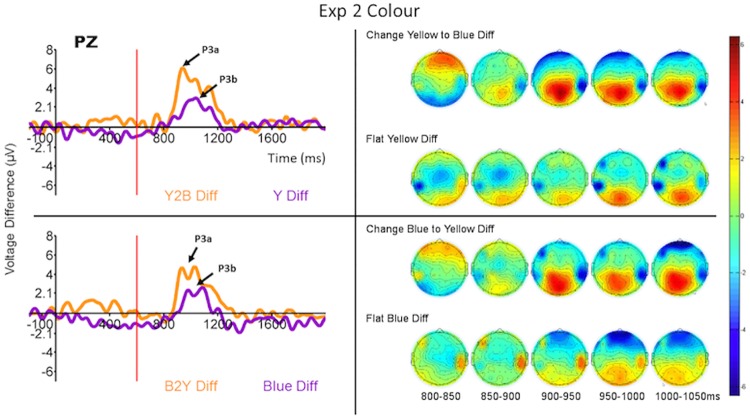
Grand average (across all subjects) ERP difference waves at Pz (P300—left) and topomaps (right) to the four run types when they were rare or common. Each panel compares the difference waves for feature-present and feature-absent conditions for run types in which the first four rings in the run were identical. (Top) Y2Blu (feature-present) compared to Y (feature-absent). (Bottom) Blu2Y (feature-present) compared to Blu (feature-absent). Time 600 ms refers to the onset of the last ring of the run. The ERPs from which the difference waves were derived are displayed for these and other selected electrode sites in S2 Fig.

**Fig 7:**
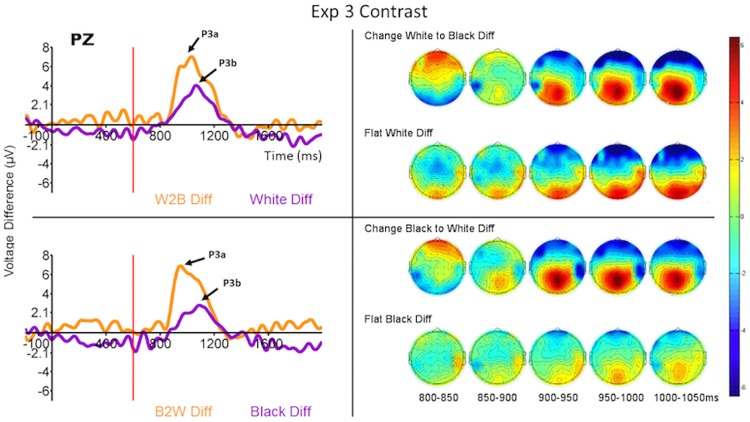
Grand average (across all subjects) ERP difference waves at Pz (P300—left) and topomaps (right) to the four run types when they were rare or common. Each panel compares the difference waves for feature-present and feature-absent conditions for run types in which the first four rings in the run were identical. (Top) W2Bla (feature-present) compared to W (feature-absent). (Bottom) Bla2W (feature-present) compared to Bla (feature-absent). Time 600 ms refers to the onset of the last ring of the run. The ERPs from which the difference waves were derived are displayed for these and other selected electrode sites in S3 Fig.

**Fig 8:**
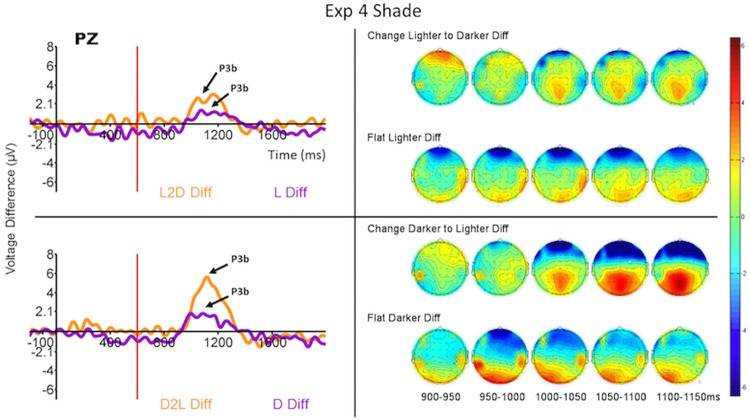
Grand average (across all subjects) ERP difference waves at Pz (P300—left) and topomaps (right) to the four run types when they were rare or common. Each panel compares the difference waves for feature-present and feature-absent conditions for run types in which the first four rings in the run were identical. (Top) L2D (feature-present) compared to L (feature-absent). (Bottom) D2L (feature-present) compared to D (feature-absent). Time 600 ms refers to the onset of the last ring of the run. The ERPs from which the difference waves were derived are displayed for these and other selected electrode sites in S4 Fig.

As in the Experiment 1 analysis, we computed the difference waves from the ERPs to the final ring of each run type when that run type was rare and when it was common. The difference waves computed from the ERPs to the last rings (onset at 600 ms relative to the onset of the first ring) in each run type are displayed in Figs 6–8 (left side) for the Pz electrode, and the ERPs themselves are displayed in S2–S4 Figs for a selection of relevant electrodes.

The overall main effect of the feature-present vs feature-absent factor in the repeated measures MANOVA was significant for P300 latency in both Experiments 2 and 3 (*F*_1,15_ = 5.04, *p* = 0.04, *η*^2^ = 0.13, and *F*_1,15_ = 7.05, *p* = 0.018, *η*^2^ = 0.19 respectively) and no other effects were significant. Similarly, the main effect of that factor was significant for P300 amplitude (*F*_1,15_ = 18.26, *p* = 0.001, *η*^2^ = 0.21, and *F*_1,15_ = 19.00, *p* = 0.001, *η*^2^ = 0.30 respectively) and again no other effects were significant. Tables 3 and 4 display the results of the planned univariate *F*-tests of P300 difference waves for the separate conditions of Experiments 2 and 3.

Figs 6 and 7 (left side) demonstrate that the P300 responses in Experiments 2 and 3 are very similar to those of the Experiment 1. In both experiments there was a clear P300-like response peaking less than 400 ms post onset of the fifth ring to the feature-present target, whereas the P300 response peaked about 500 ms post onset of the fifth ring to the feature-absent target. There was no such response when those same runs were common (S2 and S3 Figs). As in Experiment 1 the difference waves for each stimulus type are nearly indistinguishable from the ERPs to the rare runs (S2 and S3 Figs) because of the low activity generated by the common runs. The shapes of the difference waves, the latencies of the peaks of the P300 responses, and the latencies of the peaks of the difference waves differed substantially, however, between the flat (feature-absent) and change (feature-present) target runs. In general the feature-present difference waves peaked earlier and higher than the corresponding feature-absent difference waves, although there were some exceptions for individual run types (see Tables 3 and 4). We also analyzed the differences between the areas under the P300 curves (numerical integration of the curves), and the results were similar to those just detailed and so are not reported here.

In Experiment 4 the P300 responses are somewhat different (Fig 8, left side). There appears to be a P300-like response in both feature-present and feature-absent targets, though they peak later (between about 400 ms and 700 ms post onset of the fifth ring) and their amplitudes are somewhat attenuated compared to those from Experiments 2 and 3. The effect of the feature-present vs feature-absent factor in the overall MANOVA in this experiment was significant, however, for P300 amplitude (*F*_1,15_ = 8.22, *p* = 0.012, *η*^2^ = 0.06). No other effects were significant.

#### P300 topographies

In Experiments 2 and 3 the P300 response was greatest at the parietal scalp sites for both feature-present and feature-absent targets (see right sides of Figs 6 and 7 and S2, S3 and S6 Figs). In the feature-present conditions the P300 had sharpest peak at the parietal sites while the response was quickly attenuated beyond centro-parietal and occipital sites. In addition, in both Experiments 2 and 3, an early positivity was observed peaking first at the frontal then central sites about 150 ms post stimulus. In the feature-absent conditions the scalp distribution showed a similar pattern to the feature-present P300 response, but the shapes of the waves were broader and shallower. No early frontal positivity was observed to feature-absent targets. In Experiment 4 the P300 responses to both feature-present and feature-absent targets were greatest at the parietal scalp sites (Fig 8, right side, and S6 Fig). In Experiment 4, however, the P300 response was more quickly attenuated and did not extend beyond parietal sites.

These impressions were confirmed by MANOVAs for repeated measures (again collapsing across the different change and flat runs) with condition and electrode as factors (same as in Experiment 1). In both Experiments 2 and 3 the interaction between condition and electrode was significant (*F*_4,60_ = 4.32, *p* = 0.004, *η*^2^ = 0.024, and *F*_4,60_ = 2.83, *p* = 0.03, *η*^2^ = 0.018 respectively); the interactions largely reflected a slower decline in P300 amplitude away from Pz for the feature-present condition than for the feature-absent condition (S6 Fig). The planned contrast at frontal electrodes was not significant for either Experiment 2 or 3. This interaction was not significant in Experiment 4. The main effect of condition was significant for all three experiments (*F*_1,15_ = 6.19, *p* = 0.02, *η*^2^ = 0.09; *F*_1,15_ = 8.33, *p* = 0.01, *η*^2^ = 0.11; *F*_1,15_ = 7.36, *p* = 0.01, *η*^2^ = 0.14 respectively) and the main effect of electrode was significant for Experiments 3 and 4 (*F*_4,60_ = 7.6, *p* = 0.00005, *η*^2^ = 0.20; *F*_4,60_ = 8.8, *p* = 0.00001, *η*^2^ = 0.14 respectively).

### Discussion

The results of Experiments 2 and 3 constitute evidence for search asymmetry using sequentially presented visual stimuli. Our response time results were similar to those of the visual search task designed by Treisman and Souther [4], as well as to the results observed in the analogous auditory Experiment 1. Target objects containing a feature-difference that distractors lacked (feature-present condition) were identified faster than target objects that were missing a feature-difference that distractors possessed (feature-absent condition). Our P300 ERP results were also highly similar to those of Luck and Hillyard [8] for simultaneously presented visual stimuli, and to those for the auditory stimuli used in Experiment 1. These results provide evidence that the similarities observed between sequentially presented auditory and simultaneously presented visual stimuli are not unique to the auditory system; searching for targets of different feature compositions appears to use similar strategies, or at least to yield similar response time and electrophysiological results, regardless of whether the items in the arrays are visual or auditory, or are presented all at once or one at a time.

The intention of Experiment 4 was to investigate whether the sequential search asymmetry effect arose from differences in attention strategies or from differences in target-distractor discriminability. It was hypothesized that by reducing the salience of the feature difference present in the change run participants would need to focus their attention to all stimuli regardless of whether they were searching for the feature-present or feature-absent target. The complete disappearance of the asymmetry under these conditions would constitute evidence that the effect in Experiments 1–3 likely resulted from the application of different attention strategies in the two conditions [2]. Should the asymmetry, or at least some indication of it, persist, however, the effect would most likely result from differences in perceptual processing in the two conditions.

The results of Experiment 4 indicate that the asymmetry did not disappear completely. Although P300 latencies and behavioral reaction times were not significantly shorter for feature-present targets, P300 amplitudes were significantly larger for feature-present targets. Moreover, the pooled *d*’ analysis indicated that an asymmetry in discriminability was indeed present in this experiment, although overall discriminability was significantly lower than in Experiments 1–3. Thus even when participants had to pay close attention to each stimulus run, the target runs that contained the small feature difference within them were easier to discriminate from a background of stimuli with no feature difference than vice versa, implying that the asymmetry arose from a difference in the perceptual processing of the stimuli.

## Experiment 5: Roving auditory sequential search

The purpose of Experiment 5, like that of Experiment 4, was to investigate whether the sequential search asymmetry arises from a difference in attention strategy or from a difference in target discriminability. Experiment 5 was designed to augment the results of Experiment 4 by a converging operation. Considering that Experiment 1 seemed to produce the largest asymmetry effect of the first four experiments, we returned to the auditory modality for this converging operation. Some previous experiments have used a roving auditory oddball paradigm in which sequences of single simple tones occasionally change in acoustic frequency; for example, participants might hear 6 tones at 500 Hz, then 4 tones at 700 Hz, then 5 tones at 350 Hz, etc. [32–34]. We reasoned that such a roving frequency paradigm would also require participants to pay close attention to each tone run because a shift in tone frequency by itself would not reliably indicate that a particular run was the designated target run.

Thus, we used a roving-frequency version of Experiment 1 to investigate further the locus of the sequential search asymmetry effect. In this adaptation, the stimulus arrays included multiple kinds of feature-present and feature-absent stimuli consisting of several different combinations of tones. Similarly to Experiment 1, in Experiment 5 the run sequences consisted of flat runs comprised of five identical-frequency tones and change runs comprised of four identical-frequency tones plus a fifth tone of a different frequency. In order to better describe the differences between Experiments 1 and 5 we term the frequency of the first four tones of a change run and the frequency of all five tones of a flat run the “base frequency” of the run. For example, if the first four tones of a change run were at 500 Hz and the final tone at 1000 Hz the base frequency of that run would be 500 Hz. Similarly if a flat run consisted of all 5 tones at 500 Hz, the base frequency of that flat run would also be 500 Hz. Whereas in Experiment 1 the base frequencies of the change runs and flat runs used in each block were always the same, in Experiment 5 the base frequency of each run changed randomly from run to run in the overall sequence of runs. Run sequences were of the same oddball format that was used in all four of the previous experiments, where one type of run was always common and the other type of run was always rare. This meant that there were no particular tone frequencies that participants could listen for to identify the rare runs among the common runs as there were in Experiment 1; only the presence or absence of a *feature difference within a run* differentiated the rare targets from the common runs.

As in Experiment 4, participants in Experiment 5 needed to focus their attention upon each stimulus run in order to identify the targets, regardless of for which target stimulus, flat run or change run, they were listening. Unlike Experiment 4, however, the roving paradigm did maintain a salient feature difference (change in tone frequency) within the change runs, whether rare or common, like those present in Experiments 1, 2, and 3. By using this roving paradigm we expected to be able to obtain converging evidence of the locus of the sequential asymmetry effect.

### Methods

#### Participants

Data were collected initially from 23 participants. Participants were recruited via the UBC Psychology Department’s online experiment participant recruitment site and via a poster on the Department’s participant recruitment bulletin board. From 15–25 participants has been shown in previous studies in our lab and others to yield reliable EEG data for either ERP or connectivity analyses given the numbers of stimulus trials in the study design. Data collection was stopped after the indicated number of participants had been included. Two participants opted to discontinue the task prior to completion and thus these data were not included in analysis. Of the 21 remaining participants, data from three participants were excluded due to extreme behavioral measures (hit rate, false alarm rate or reaction time z-scores < -2.5, > 2.5, or > 2.5 respectively) for either feature-present or feature-absent conditions, or for excessive noise in the EEG recordings. Thus the analysis to be described is based on 18 participants (13 female, age 18 to 31 years, mean age 22.3). All aspects of the experimental protocol, including the recruitment and consent procedures, were approved by the University of British Columbia Behavioural Research Ethics Board in accordance with the provisions of the World Medical Association Declaration of Helsinki. Participants gave written informed consent by reading and signing the approved consent document, and were offered monetary compensation ($10/hr) for their participation. All participants but one were right-handed and no participants reported hearing or neurological difficulties.

#### Stimuli

**Fig 9:**
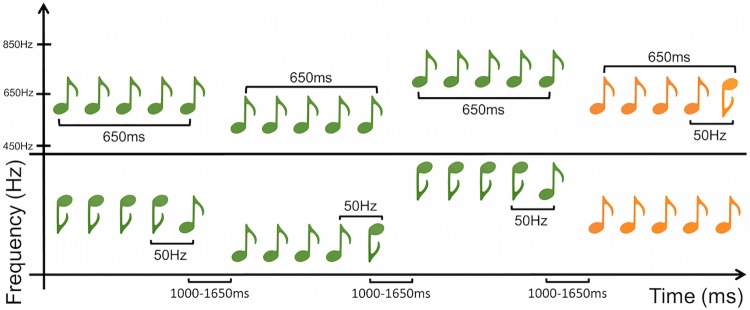
Experiment 5 sequence design. Participants each heard two types of extended sequences of runs, flat common/change rare (top) and change common/flat rare (bottom). Flat stimuli varied in base frequency, and change stimuli could be either change up or change down from the varied base frequency, as described in the Methods section. Common runs are in green, rare runs are in orange.

As in Experiment 1, we generated 50-ms duration pure tones with 7.5 ms onset and offset ramps, using the ascending first half of a Hann window for the onset and the descending second half of the Hann for the offset, and using a custom MATLAB (MathWorks Natick USA) script. Individual tones in a run were stitched together using Audacity (Sourceforge). Tones were presented binaurally at 70 dB via insert earphones (EAR 3A) in a sound-attenuating chamber. Stimuli were presented and responses registered using Presentation software (Neurobehavioral Systems Berkeley CA USA). Auditory stimuli consisted of two types of five-tone runs *flat runs* and *change runs* as in Experiment 1. In Experiment 5 however flat runs and change runs were built from combinations of nine different frequencies that ranged from 450 Hz to 850 Hz and were separated by 50 Hz steps. There were seven different flat runs which consisted of 500 Hz, 550 Hz, 600 Hz, 650 Hz, 700 Hz, 750 Hz, and 800 Hz tones. There were 14 different change runs where the first four tones were one of the seven frequencies used to build the flat runs, and the fifth tone was either one step higher or one step lower than the previous four. For example there were two change runs that began with 500 Hz tones, but one change run ended with a 450 Hz tone whereas the other ended with a 550 Hz tone (see Fig 9 for a detailed illustration). Each tone was 50 ms in duration and successive tones in a run were separated by 100 ms of silence. Each run lasted 650 ms from the onset of the first tone to the offset of the last tone. Intervals between the offset of the final tone of a given run and the onset of the first tone of the next run varied randomly from 1000 ms to 1650 ms.

#### Procedure

Once participants were comfortably seated in the sound-attenuating chamber they were familiarized with the stimuli and protocol. Each participant heard eight extended oddball sequences of tone runs (about 4.5 mins per sequence) in randomized order per subject. Each sequence began with 30 instances of common runs. From then on the rare run was presented on a random 20% of occasions among 80% common runs. In each sequence rare runs were heard between 18 and 30 times. There were always at least two common runs before and after each rare run. The base frequency of each run was randomly selected from the range of stimulus frequencies (described above) with the exception that each run, common or rare, was never identical in base frequency to the run that preceded it. The eight sequences of runs consisted of two types that were each repeated four times: (1) common flat/rare change; (2) common change/rare flat. Participants were instructed to click a mouse every time they heard a pattern oddball (rare run) and their response times were recorded. It should be noted that it was made clear to participants that they were only to respond when they heard a change in the global pattern, i.e. a rare run, *not* every time they heard a tone that differed from the previous tone.

#### EEG recording and ERP extraction

EEG recording and ERP extraction were identical to that of Experiments 1–4. The latency window from which the peak amplitudes were determined was 200 to 1000 ms after the onset of the last tone.

#### ERP components and measurement

Data analyses and terminology were identical to those used for Experiments 1–4, except that in Experiment 5 there were many more different flat and change run stimuli than in the previous experiments, and thus there were many fewer trials of each specific stimulus run. In other words whereas all of the stimuli in Experiments 1 through 4 comprised four different specific runs, two change and two flat, Experiment 5 consisted of 21 different specific run stimuli, seven flat and 14 change. These 21 different run stimuli were distributed roughly evenly among each block depending on block type, although in feature-present-target blocks there were 80% flat runs and in feature-absent-target blocks there were 80% change runs, which is the same across all five experiments. Having fewer trials of each run meant that all change runs and all flat runs needed to be collapsed within each condition so that the ERP and behavioral analyses would be based on sufficient trials per condition. Collapsing across specific run stimuli prevented an analysis of the P300 difference wave generated by subtracting the ERP to the exact same run when it was common from that when it was rare, as was done in Experiments 1 through 4. Instead the P300 difference wave generated by subtracting the ERPs to all flat runs when they were common from those when they were rare, and all those for change runs when they were common from those for when they were rare, regardless of their specific tonal composition, was used to determine the ERP latency and amplitude results of Experiment 5.

### Results

#### Behavioral results

Table 6 summarizes the response time results of the experiment. The response times to correctly detect the rare target run before the onset of the final tone of the following non-target run were calculated from the onset of the fifth tone in the run. In contrast to Experiments 1, 2, and 3, and consistent with Experiment 4, response times to feature-present and feature-absent targets were not significantly different by dependent means *t*-test. False alarm responses to common (non-target) runs were very infrequent, about 1.6% overall. As for the previous experiments, the pooled *d*’ analysis in Experiment 5 indicated that feature-present targets were significantly more discriminable than were feature-absent targets from their surrounding sequences (Table 2).

**Table 6:** Experiment 5 RT and electrophysiological results.

Target Run	RT (ms)	P3 Lat (ms)	P3 Am (μV)
**Change**	552	487	8.3
**Flat**	571	523	7.1
**Difference**	20	35	1.2
***p***	0.14	0.22	0.006*
***t(17) (es)***	1.53 (N/A)	1.26 (N/A)	3.06 (0.57)

Results are based on the average of all individual differences between conditions. Significant differences are indicated with an asterisk (*). Effect size (*es* = √(*t*^2^/(*t*^2^ + *df*))) estimates are only included for significant differences.

#### P300 ERP results at Pz

**Fig 10:**
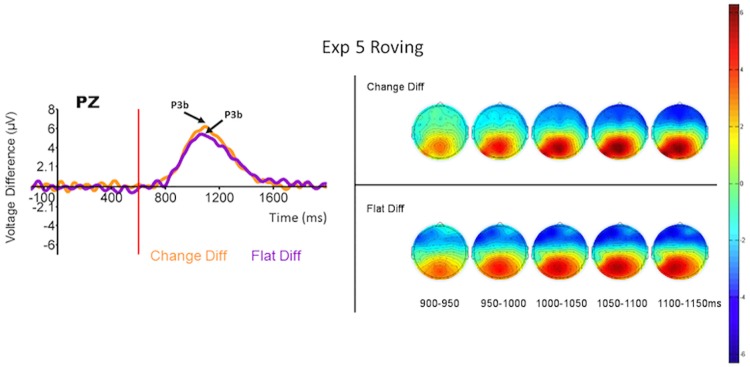
Grand average (across all subjects) ERP difference waves at Pz (P300—left) and topomaps (right) to the feature-present (change) and feature-absent (flat) conditions. Time 600 ms refers to the onset of the last tone of the run The ERPs from which the difference waves were derived are displayed for these and other selected electrode sites in S5 Fig.

As in Experiment 1 we computed the difference waves from the ERPs to the final tone of each run type when that run type was rare and when it was common. In contrast to Experiment 1 these difference waves were not computed for each specific run but only for run type as described above. The respective difference waves comparing the ERPs to the last tones (onset at 600 ms relative to the onset of the first tone) to the same run type when it was rare and when it was common, are displayed in Fig 10 (left side) for the Pz electrode, and the ERPs themselves are displayed in S5 Fig for a relevant subset of electrodes. The difference waves to the feature-present targets peak later (approximately 500 ms post onset of the fifth tone) compared to those from Experiments 1–3, and at around the same time as those to the feature-absent targets in Experiments 1–3 and in the present experiment. Whereas the peak latencies of feature-present and feature-absent P300s are not significantly different, the amplitude of the feature-present P300 is significantly larger than that of the feature-absent P300 (Table 6).

#### P300 topographies

The P300 response in Experiment 5 was greatest at the parietal scalp sites for both feature-present and feature-absent targets (right side of Fig 10 and S5 and S6 Figs). In the feature-present conditions the P300 had sharpest peak at the parietal sites while the response was quickly attenuated beyond centro-parietal and occipital sites. In the feature-absent conditions the scalp distribution showed a similar pattern to the feature-present P300 response, where the amplitude was also largest at parietal sites and quickly attenuated beyond centro-parietal and occipital sites. No early frontal positivity was observed in either condition. These impressions were confirmed by a MANOVA for repeated measures in which only the main effect of electrode was significant (*F*_4,76_ = 41.86, *p* < 0.000001, *η*^2^ = 0.53).

### Discussion

The results suggest that the differences between feature-present and feature-absent conditions in RTs and P300 latencies that were observed in Experiments 1, 2, and 3 were not found in Experiment 5. If search asymmetry is defined simply as a difference in search times to these two conditions, as in [4], then the results of Experiment 5 constitute an elimination of the search asymmetry. An asymmetry does persist, however, when considering the difference in accuracy between the two conditions. Although the roving-frequency task was more difficult to perform than were the tasks in Experiments 1–3, the pooled *d*’ analysis indicated that a discriminability difference between the two conditions was present. Our ERP results also suggest a persistence of the asymmetry: the amplitude of the feature-present target P300 was significantly larger than that of the feature-absent target P300. Taken together these results suggest that this adaptation of the roving sequential search paradigm did eliminate some typical signs of a search asymmetry, namely the shorter response time and P300 latency to the feature-present target. The asymmetry persisted, however, in terms of detection accuracy and P300 amplitude. These results will be further interpreted in the context of all five experiments in the General Discussion.

## General discussion

Taken together the results of Experiments 1, 2, and 3 provide convincing evidence that important properties of search through sequentially presented auditory arrays (Experiment 1) and visual arrays (Experiments 2 and 3) resemble those of search through static visual arrays [4] and analogous, very briefly presented, auditory arrays [16]. In particular, searches through the sequentially-presented arrays exhibited the same kind of asymmetry of response that characterizes searches through static visual arrays: search times [4] and P300 latencies [8] to identify highly discriminable targets were significantly faster in the feature-present condition than in the feature-absent condition. These results suggest that the strategies used to search through sequentially presented stimuli may be similar in some ways to the strategies used to search through static visual arrays.

Experiments 4 and 5 also provide evidence of an asymmetry. Although not the kind typically associated with classic search asymmetry, the discriminability asymmetry resembles that reported by Cusak and Carlyon [16]. Whereas the reaction times and P300 latencies were not different between conditions, the accuracy (*d’* values) and P300 amplitudes were significantly different between conditions, suggesting that, despite the more challenging tasks, the feature-present target was still easier to correctly identify than was the feature-absent target. Considering that *d’* values were significantly different between conditions across all five experiments, it would appear that the feature-present target was consistently more easily identified than the feature-absent target, irrespective of the time it took to identify these targets. It is possible that reaction time differences may only be associated with the asymmetry in the context of certain task demands.

This interpretation is consistent with the ERP results. It would appear that the different task demands of identifying the feature-present and feature-absent targets do in fact elicit different contributions of these neural responses in the first three experiments. The feature-present condition seems to elicit characteristics of both the P3a and P3b subcomponents, namely the sharp early peak of the P3a but with a slow return to baseline, taking approximately 400 ms, which is characteristic of the P3b. This is consistent with the current understanding of the conditions under which the P3a and P3b are typically observed: the feature-present target is not only a task-relevant oddball (P3b) but also contains a salient perceptual oddball that is both infrequent and unpredictable (P3a). We will use the study design of Experiment 1 (auditory) to illustrate this in detail. In every longer sequence of runs the target run was heard at a 20% probability. Given that each run consisted of 5 tones, in any feature-present sequence of, e.g., 100 trials, participants would hear on average 480 instances of the same tone (e.g., 500 Hz if the common run was flat low), and only 20 instances of the other possible tone (e.g., 1000 Hz if the common run was flat low). Not only were these infrequent and unpredictable tones also highly perceptually salient given the tonal context, they also indexed the target stimulus. Therefore, it is not surprising that the feature-present target P300 response is characteristic of both the P3a and the P3b, because the target shared properties with the types of stimuli with which these responses are typically associated. This same logic can also be applied to the results of Experiments 2 and 3.

In contrast, the broad and relatively flat shapes of the P300 ERPs in response to the feature-absent targets, and their scalp topographies, suggest that they are mostly P3b ERPs, which is also consistent with the literature [9,10]. In contrast to the feature-present target, the rare feature-absent target does *not* contain a highly perceptually salient feature change, nor is there any perceptually infrequent or unpredictable stimulus indexing the feature-absent target. It is simply made up of five of the most frequent stimuli (tones or rings) instead of only containing four of them. Therefore it is not surprising that the feature-absent target does not elicit a P3a.

The frequent change run in this condition, however, *does* contain a relatively infrequent, perceptually salient, different fifth tone (or ring) at the end of each run, so one might think that each change run might produce a P3a response. To make this clear, using the previous example (Experiment 1), in any block of 100 trials where the feature-absent run was the rare target, participants would hear on average 420 instances of the same tone (e.g., 500 Hz if the common run was change up) and 80 instances of the other tone (e.g., 1000 Hz if the common run was change up). Though the perceptually-salient stimulus in this case is also relatively infrequent in the context of the feature-absent condition, *it is nonetheless highly predictable*, occurring on 80% of runs, and therefore should not generate a P3a. This is indeed what was found in the present study, as can be seen in S1–S3 Figs where the responses to the common change runs in the feature-absent condition are generally around baseline, and virtually indistinguishable from the responses to the common flat runs in the feature-present condition, which do not contain such perceptually-salient stimuli. Taken together, every common (change) run of the feature-absent condition contains an infrequent perceptually-salient but predictable stimulus that doesn’t elicit a P3a subcomponent, whereas every rare target (change) run of the feature-present condition contains an infrequent, perceptually-salient, unpredictable stimulus (that also serves to identify the target) that does elicit a P3a.

In Experiment 5, however, neither the feature-present nor the feature-absent targets appear to elicit a substantial P3a subcomponent, only a P3b. This is consistent with the above characterization of the P3a and P3b in the context of the roving paradigm. Because the tonal context is constantly changing in both conditions, the different fifth tone of the change runs is not unique or salient in the feature-present condition and therefore will not elicit a P3a. The absence of the P3a could account for the lack of P300 peak latency and reaction time differences between the two conditions.

The distractor-evoked P3a [9,10] does typically peak much earlier than the target-evoked P3b, which is consistent with what we observed in Experiments 1, 2, and 3. These different peak latencies also could account for the reaction time differences in the first three experiments.

One difficulty for this interpretation is that in general P300 latencies and behavioral reaction times have been considered to be independent [35–37]. There has been less investigation, however, into the relationship between the latencies of the different subcomponents of the P300 and reaction times. As far as we know ours is the first study to explicitly describe a relationship between the presence of a strong P3a subcomponent in ERPs to targets, and shorter behavioral response times to those targets. One recent study did demonstrate, however, that summed P3a and P3b subcomponents in response to targets can be dissociated, at least in an RSVP attention blink paradigm [38], although the relationship of these separate components to response times was not studied. Nonetheless the separated-out target-evoked P3a subcomponent had a shorter peak latency than did the target-evoked P3b, similar to the typical relationship between the distractor-evoked P3a and the target-evoked P3b, and also similar to the relationship between feature-present and feature-absent P300 responses in our Experiments 1–3.

Although they didn’t characterize their P300 ERPs in terms of their subcomponents, Luck and Hillyard [8] reported that the P300 peak latency to feature-present targets in their visual search paradigm was roughly constant and shorter (at around 520 ms) than that to feature-absent targets, which increased linearly with distractor set size (from around 600 ms to about 750 ms for distractor sets of 4 and 12 elements respectively). Similarly, response time on target-present trials was constant and shorter for feature-present targets (at around 680 ms), and increased linearly with distractor set size for feature-absent targets (from around 740 ms to around 1050 ms for sets from 4 to 12 elements). Moreover, the P300 to feature-present targets was much sharper than the P300 to the feature-absent targets; indeed the P300 to the feature-present targets closely resembled the P3a-dominated P300 we saw to our rare change targets in Experiments 1–3, whereas the P300 to the feature-absent targets resembled that to our rare flat targets in Experiments 1–3. Thus P300 peak latencies are indeed related to response times under some conditions, especially when a P3a subcomponent is prominent, as in our Experiments 1–3 and in Luck and Hillyard’s [8] visual search experiments.

It has been shown in 3-stimulus (common standards, rare targets, and rare salient distractors) visual and auditory oddball paradigms that both the peak latency and the morphology of the P300 ERP are sensitive to the discriminability of the target [11,39]. Comerchero and Polich [39] compared P300 responses to infrequent target stimuli that were either easy or difficult to discriminate from the more frequent standards in both auditory and visual oddball tasks. The P300 responses to easy targets were also earlier, sharper and larger than the P300 responses to the targets that were difficult to discriminate. Those results are consistent with what we found in Experiments 1–3 and with the P300 peak latency and morphology differences observed in the experiments of Luck and Hillyard [8]. Our scalp topographies, however, differ slightly from what Comerchero and Polich observed, particularly in Experiments 2 and 3. Consistent with our Experiment 1, Comerchero and Polich found that auditory P300 responses were stronger at the frontal electrodes in the easy condition but very weak at frontal sites in the difficult condition. In the visual task, however, Comerchero and Polich [11,39] found strong P300 responses at frontal electrodes for both difficult and easy conditions, whereas in our Experiments 2–5 the P300 responses were weak at frontal electrodes in both feature-present and feature absent conditions. The (visual) scalp topographies observed by Luck and Hillyard, however, were more similar to those of our (auditory) Experiment 1, and to the auditory results of Comerchero and Polich than to the visual results of Comerchero and Polich [11,39]. Taken together, this strongly suggests that P300 scalp topographies are highly sensitive to stimulus context; perhaps we’re observing a slight variation in underlying attention networks due to the complexity of our visual stimuli in Experiments 2 and 3. Despite these topographic differences, therefore, the latency and morphological similarities at parietal electrodes still strongly suggest a greater contribution of the P3a subcomponent in the easier feature-present condition than in the more difficult (but still fairly easy, viz. the large *d*’s) feature-absent condition for Experiments 1–3. An infrequent unpredictable target that is easy to discriminate from standards is also likely to be highly perceptually salient and thus might elicit a stronger P3a subcomponent than would a target that is very similar to the standards. This could be one reason for the difference in P300 peak latencies and morphologies found by Comerchero and Polich [11,39] for easy and difficult tasks, as we argue is also true for the differences between feature-present and feature-absent responses in our Experiments 2 and 3.

In the absence of a prominent P3a subcomponent that could produce a reaction time difference in Experiment 5, the P300 amplitude (at Pz) and *d*’ differences between conditions suggest that there still exists a perceptual asymmetry, meaning that the feature difference present in the change runs renders the feature-present targets perceptually more discriminable than the feature-absent targets. A perceptual asymmetry indexed by a difference in accuracy measures between feature-present and feature-absent conditions was also found earlier by Cusak and Carlyon [16]. They concluded that FIT was the most appropriate explanation for their results. This also appears to be the case for the present study, as differential activation of cortical feature maps would still apply to serial presentation of a stimulus array. This has been mentioned in the context of the RSVP literature as well [40]. Therefore, despite both feature-present and feature-absent targets in all five studies being highly identifiable as evidenced by the high *d*’ values, the addition of the highly salient feature difference of the change run in Experiments 1–3 may have engaged the P3a attention orienting network, which is a faster network than the P3b context updating network. This then would be responsible for the faster responses to the feature-present target than to the feature-absent target in those experiments.

Overall the results of this study suggest that the search asymmetry in our sequential paradigm likely arises from differences in the perceptual processing of the feature-present and feature-absent targets, possibly reflecting differences in activations of sensory feature maps, as the differences in *d*’ values between conditions across all five experiments would indicate. They also suggest, however, that the task demands of the experiment influence which attention strategies are used to perform the task. When the target contains a rare, unpredictable, and perceptually salient feature, a diffuse attention strategy may be all that is necessary to perform the task, as the stimulus-driven attention-orienting P3a network could be automatically activated by that stimulus even if it indexes a target. On the other hand when a target does not contain a highly salient and perceptually salient feature, a focused attention strategy is required to correctly identify the stimulus.

## Supporting information

S1 Fig ERPs for the four legend-indicated comparisons for Experiment 1. (PDF)

S2 Fig ERPs for the four legend-indicated comparisons for Experiment 2. (PDF)

S3 Fig ERPs for the four legend-indicated comparisons for Experiment 3. (PDF)

S4 Fig ERPs for the four legend-indicated comparisons for Experiment 4. (PDF)

S5 Fig ERPs for the two legend-indicated comparisons for Experiment 5. (PDF)

S6 Fig Interactions of feature present/absent condition with midline electrode location for P300 amplitudes for indicated experiment. FP (blue) indicates feature-present condition; FA (red) indicates feature-absent condition.
(PDF)
