# Supplementary material for: Sequential search asymmetry: Behavioral and psychophysiological evidence from a dual oddball task
Source: PLoS One. 2017 Mar 9;12(3):e0173237. doi: 10.1371/journal.pone.0173237 (PMC5344355; doi:10.1371/journal.pone.0173237)
Supplement: S1 Fig — (PDF) [file pone.0173237.s001.pdf]

Supplementary Information – S1 Fig

Sequential search asymmetry: Behavioral and psychophysiological evidence from a dual oddball task

Elizabeth G. Blundon, Samuel P. Rumak, Lawrence M. Ward*

- **Correspondence:** Lawrence M. Ward: lward@psych.ubc.ca

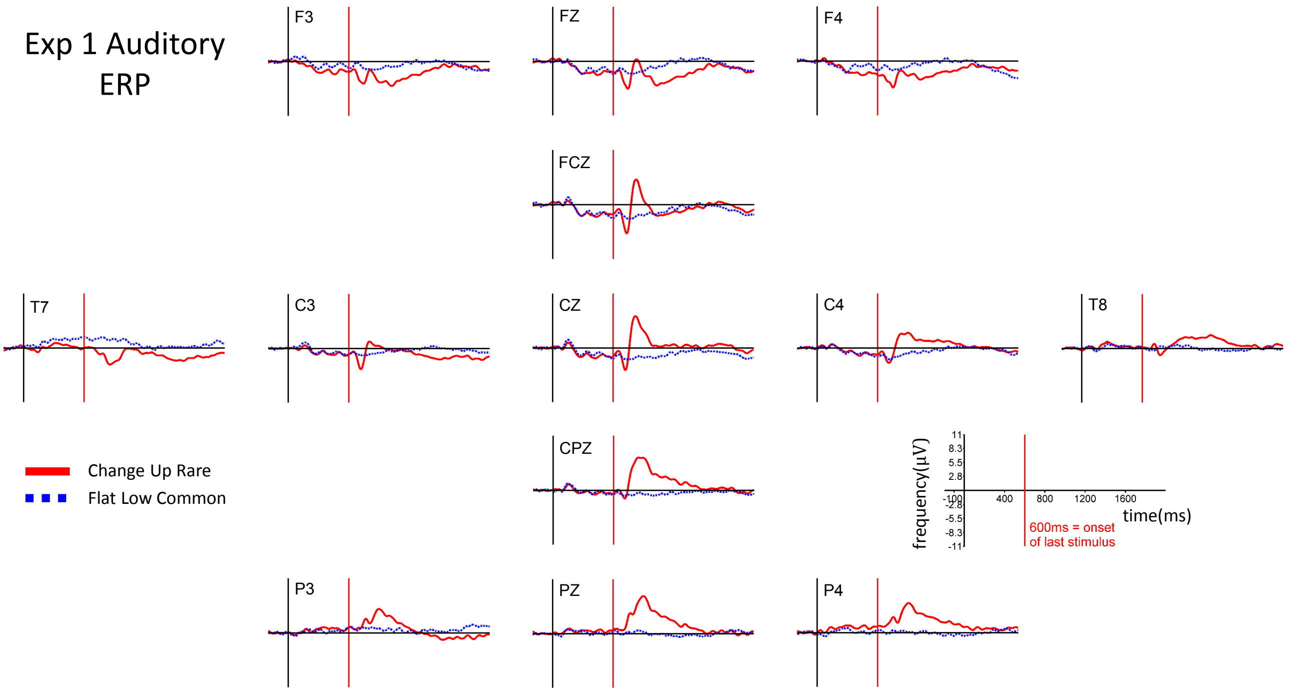
Exp 1 Auditory ERP
F3
FZ
F4
FCZ
T7
C3
CZ
C4
T8
Change Up Rare
Flat Low Common
CPZ
frequency(µV)
11
8.3
5.5
2.8
-100
-2.8
-5.5
-8.3
-11
400
800
1200
1600
time(ms)
600ms = onset of last stimulus
P3
PZ
P4

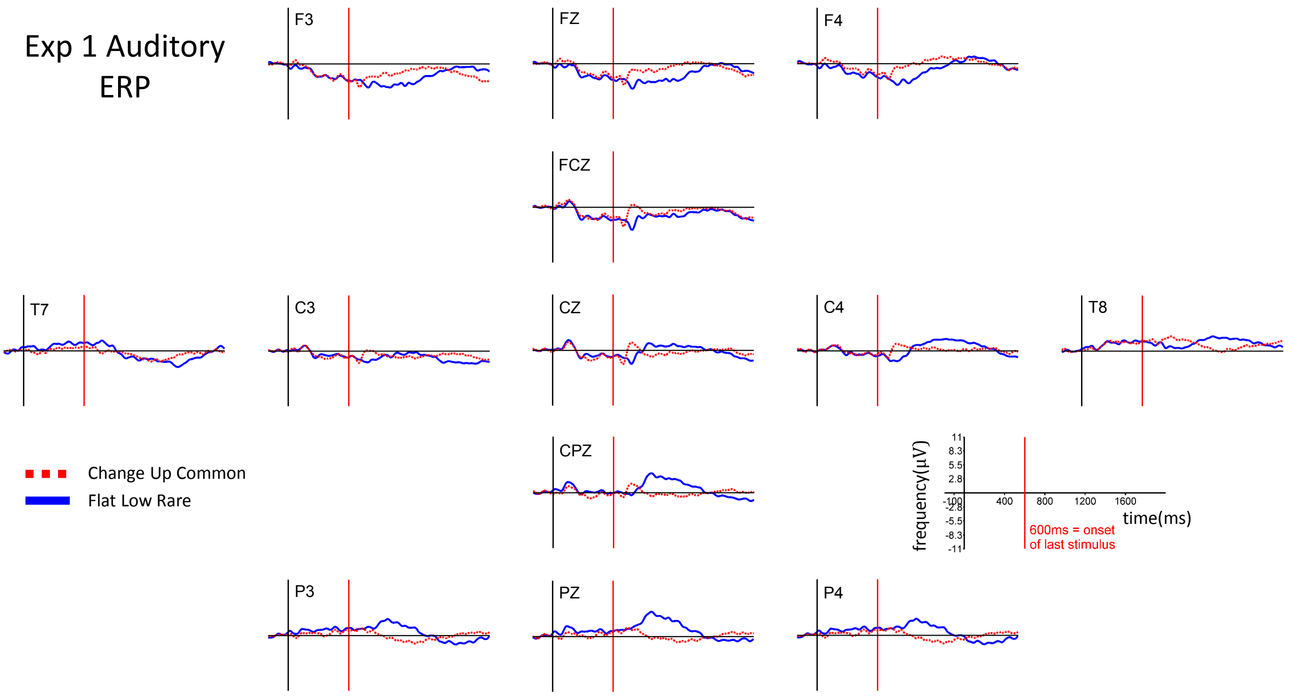
Exp 1 Auditory ERP
F3
FZ
F4
FCZ
T7
C3
CZ
C4
T8
Change Up Common
Flat Low Rare
CPZ
frequency(µV)
11
8.3
5.5
2.8
-100
-2.8
-5.5
-8.3
-11
400
800
1200
1600
time(ms)
600ms = onset of last stimulus
P3
PZ
P4

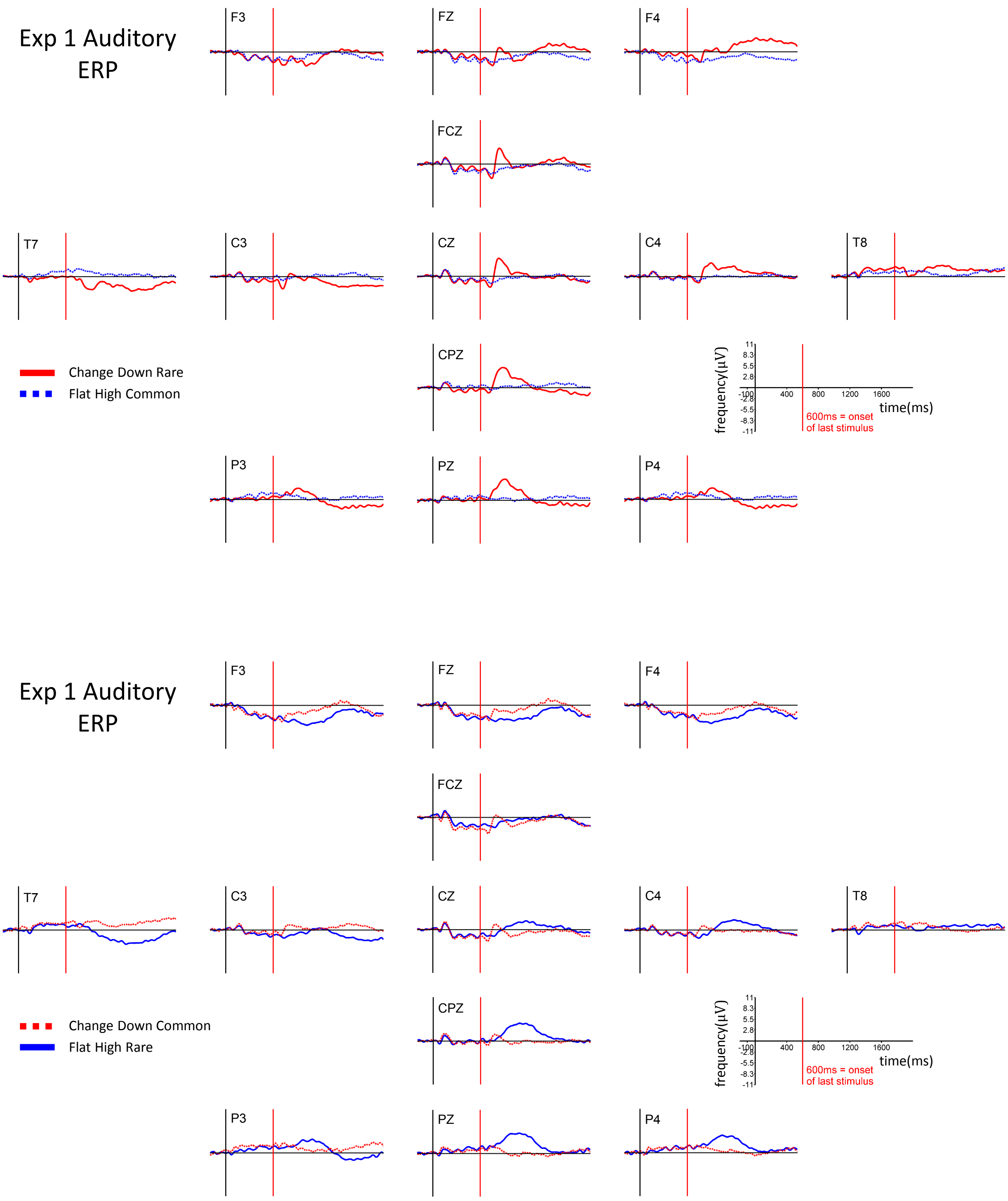

S1 Fig. ERPs for the four legend-indicated comparisons for Experiment 1.
