# Supplementary material for: Sequential search asymmetry: Behavioral and psychophysiological evidence from a dual oddball task
Source: PLoS One. 2017 Mar 9;12(3):e0173237. doi: 10.1371/journal.pone.0173237 (PMC5344355; doi:10.1371/journal.pone.0173237)
Supplement: S2 Fig — (PDF) [file pone.0173237.s002.pdf]

Supplementary Information – S2 Fig

Sequential search asymmetry: Behavioral and psychophysiological evidence from a dual oddball task

Elizabeth G. Blundon, Samuel P. Rumak, Lawrence M. Ward*

*** Correspondence:** Lawrence M. Ward: lward@psych.ubc.ca

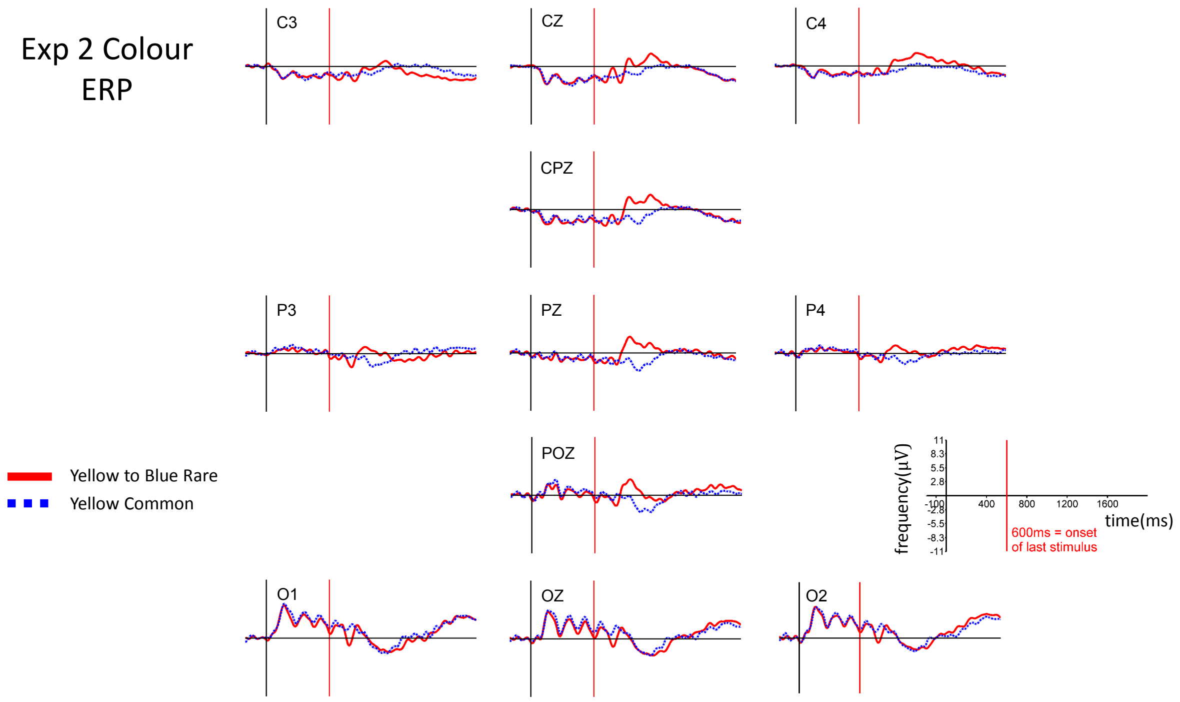
Exp 2 Colour ERP
C3
CZ
C4
CPZ
P3
PZ
P4
POZ
O1
OZ
O2
Yellow to Blue Rare
Yellow Common
frequency(μV)
11
8.3
5.5
2.8
-100
-2.8
-5.5
-8.3
-11
400
800
1200
1600
time(ms)
600ms = onset of last stimulus

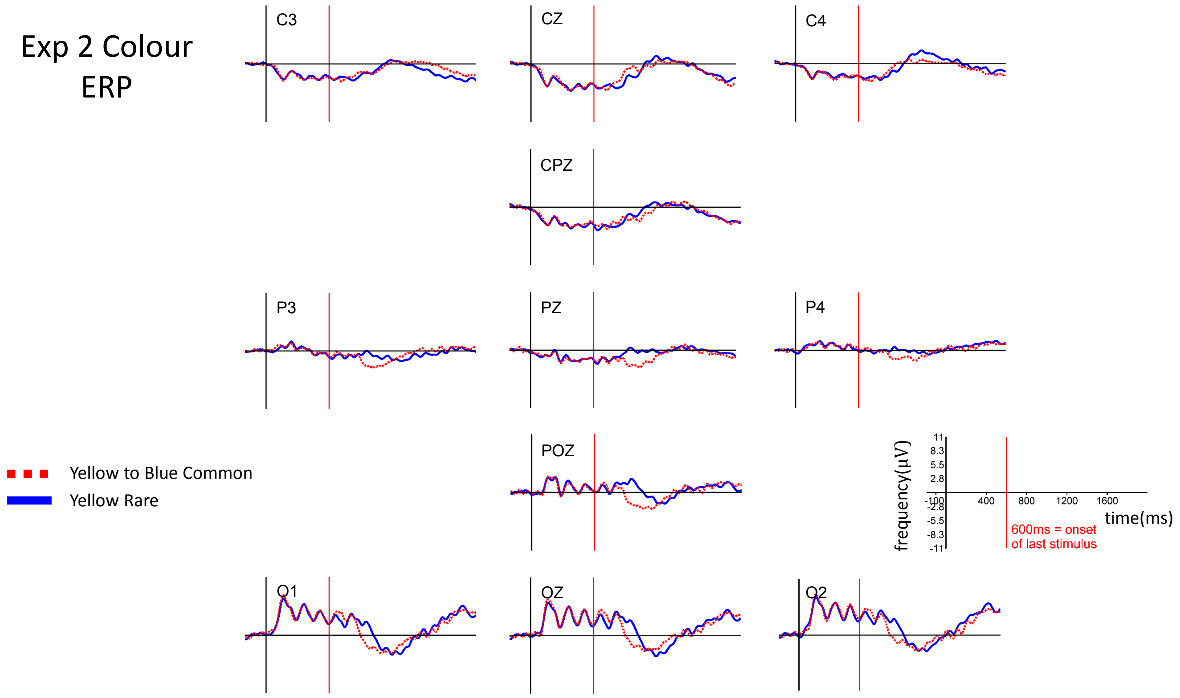
Exp 2 Colour ERP
C3
CZ
C4
CPZ
P3
PZ
P4
POZ
O1
OZ
O2
Yellow to Blue Common
Yellow Rare
frequency(μV)
11
8.3
5.5
2.8
-100
-2.8
-5.5
-8.3
-11
400
800
1200
1600
time(ms)
600ms = onset of last stimulus

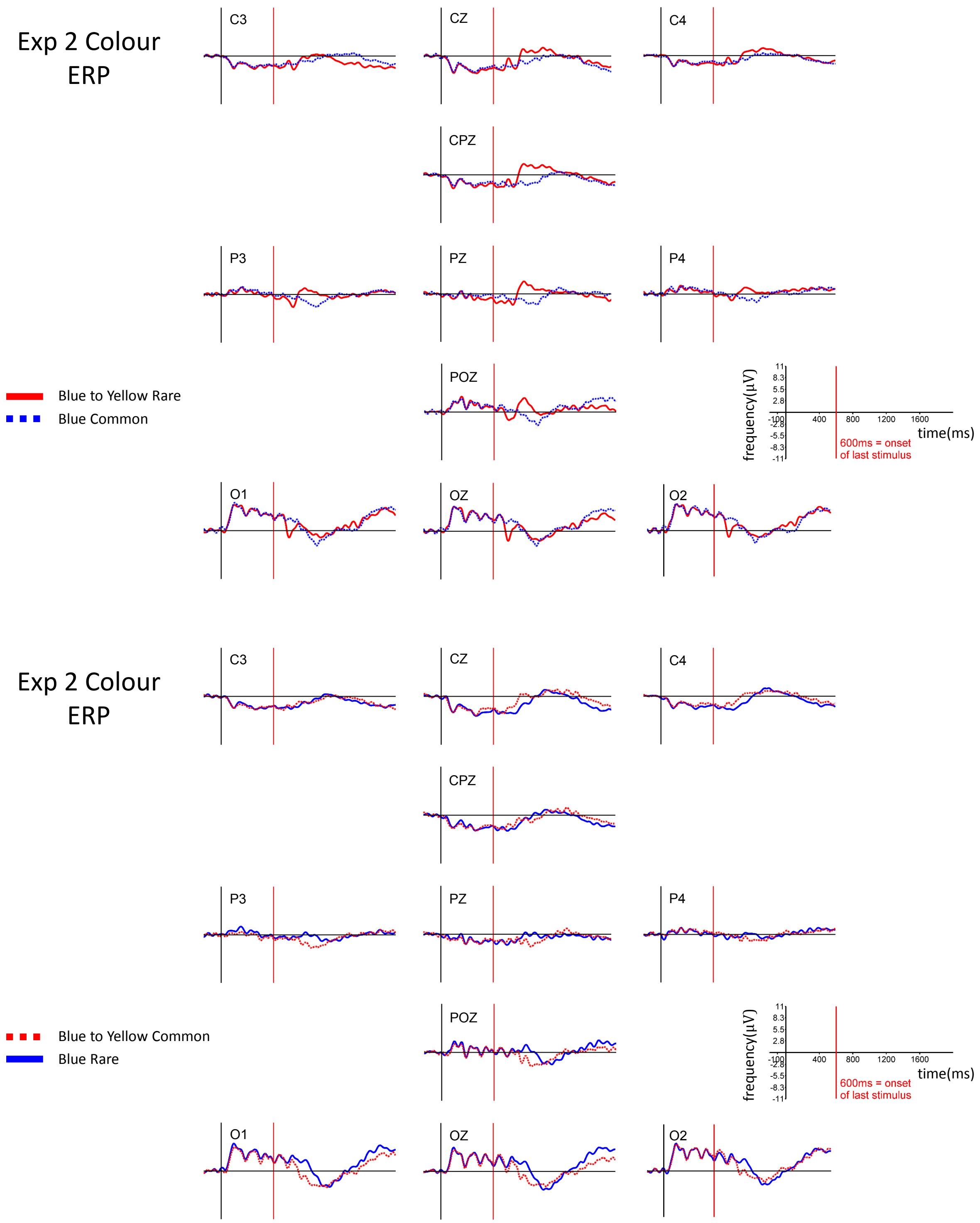

S2 Fig. ERPs for the four legend-indicated comparisons for Experiment 2.
