# Supplementary material for: Sequential search asymmetry: Behavioral and psychophysiological evidence from a dual oddball task
Source: PLoS One. 2017 Mar 9;12(3):e0173237. doi: 10.1371/journal.pone.0173237 (PMC5344355; doi:10.1371/journal.pone.0173237)
Supplement: S4 Fig — (PDF) [file pone.0173237.s004.pdf]

Supplementary Information – S4 Fig

Sequential search asymmetry: Behavioral and psychophysiological evidence from a dual oddball task

Elizabeth G. Blundon, Samuel P. Rumak, Lawrence M. Ward*

*** Correspondence:** Lawrence M. Ward: lward@psych.ubc.ca

Exp 4 Shade ERP

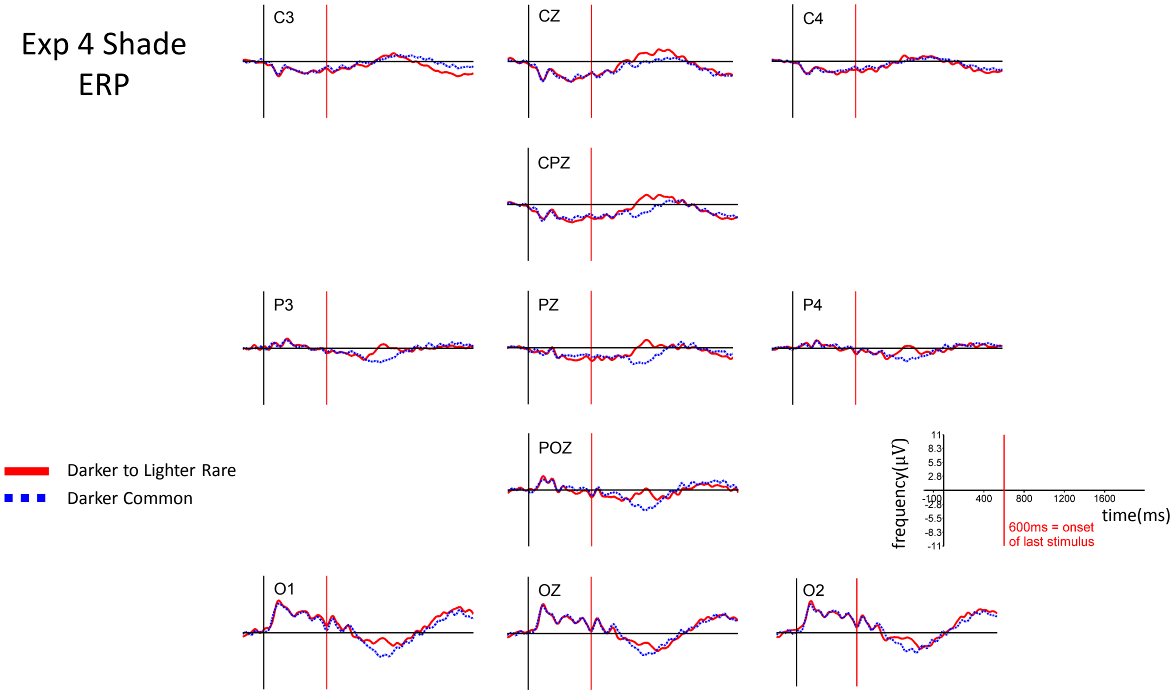

Exp 4 Shade ERP

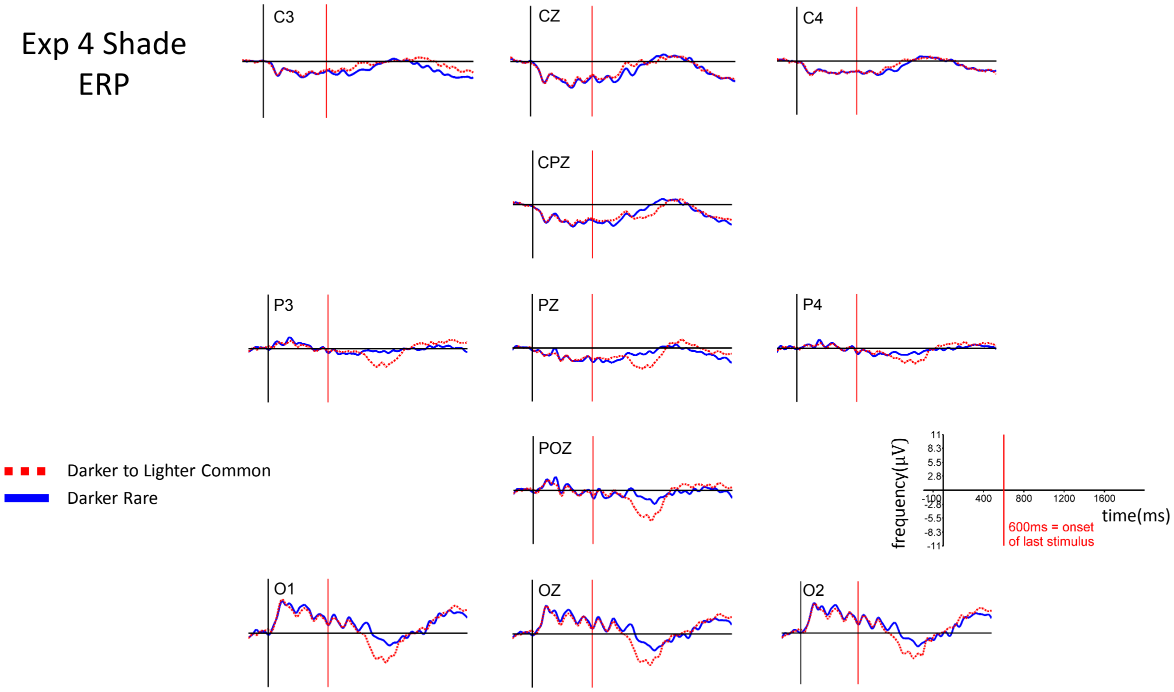

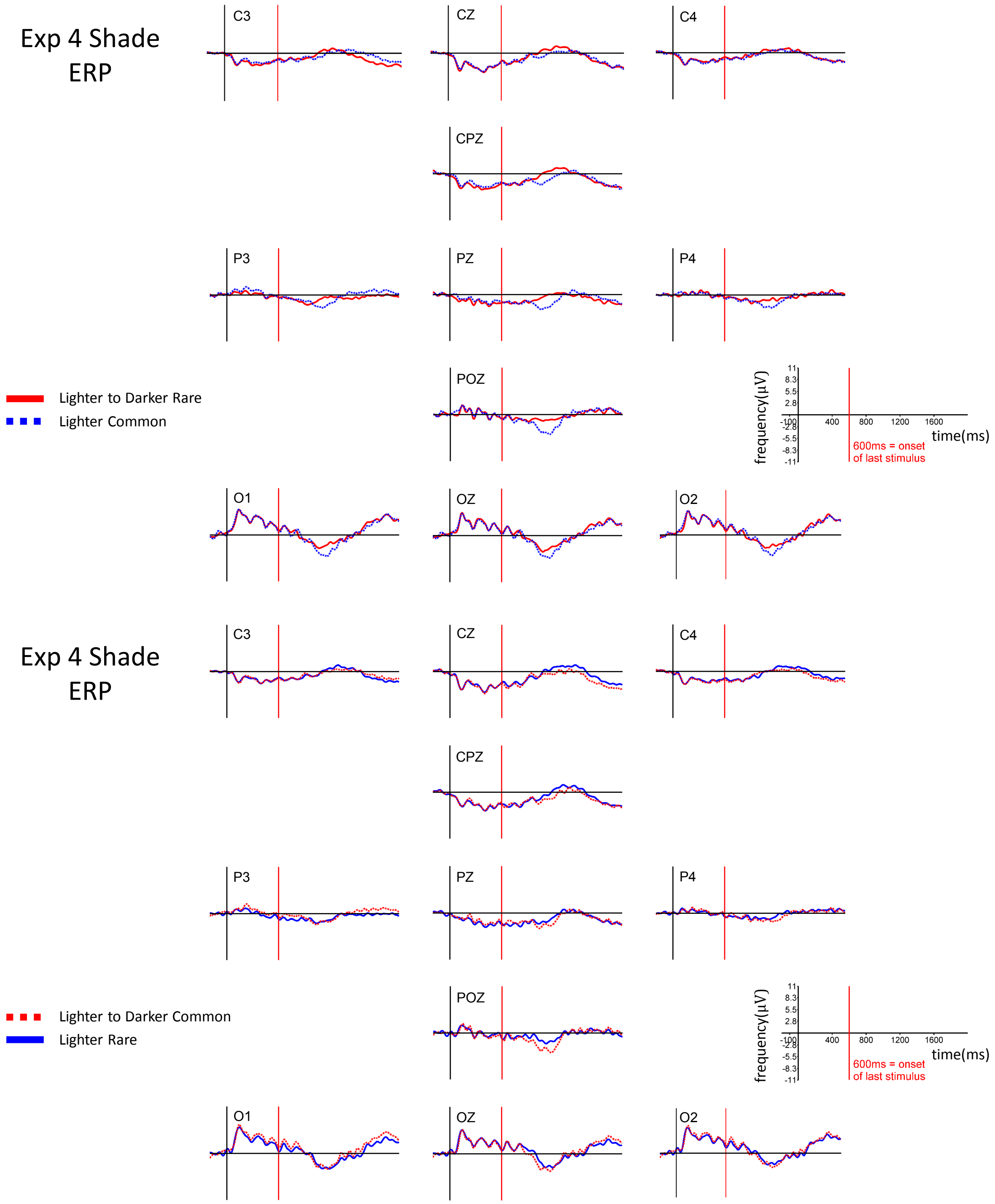

S4 Fig. ERPs for the four legend-indicated comparisons for Experiment 4.
