# Supplementary material for: Sequential search asymmetry: Behavioral and psychophysiological evidence from a dual oddball task
Source: PLoS One. 2017 Mar 9;12(3):e0173237. doi: 10.1371/journal.pone.0173237 (PMC5344355; doi:10.1371/journal.pone.0173237)
Supplement: S6 Fig — FP (blue) indicates feature-present condition; FA (red) indicates feature-absent condition. (PDF) [file pone.0173237.s006.pdf]

Supplementary Information – S6 Fig

Sequential search asymmetry: Behavioral and psychophysiological evidence from a dual oddball task

Elizabeth G. Blundon, Samuel P. Rumak, Lawrence M. Ward*

*** Correspondence:** Lawrence M. Ward: lward@psych.ubc.ca

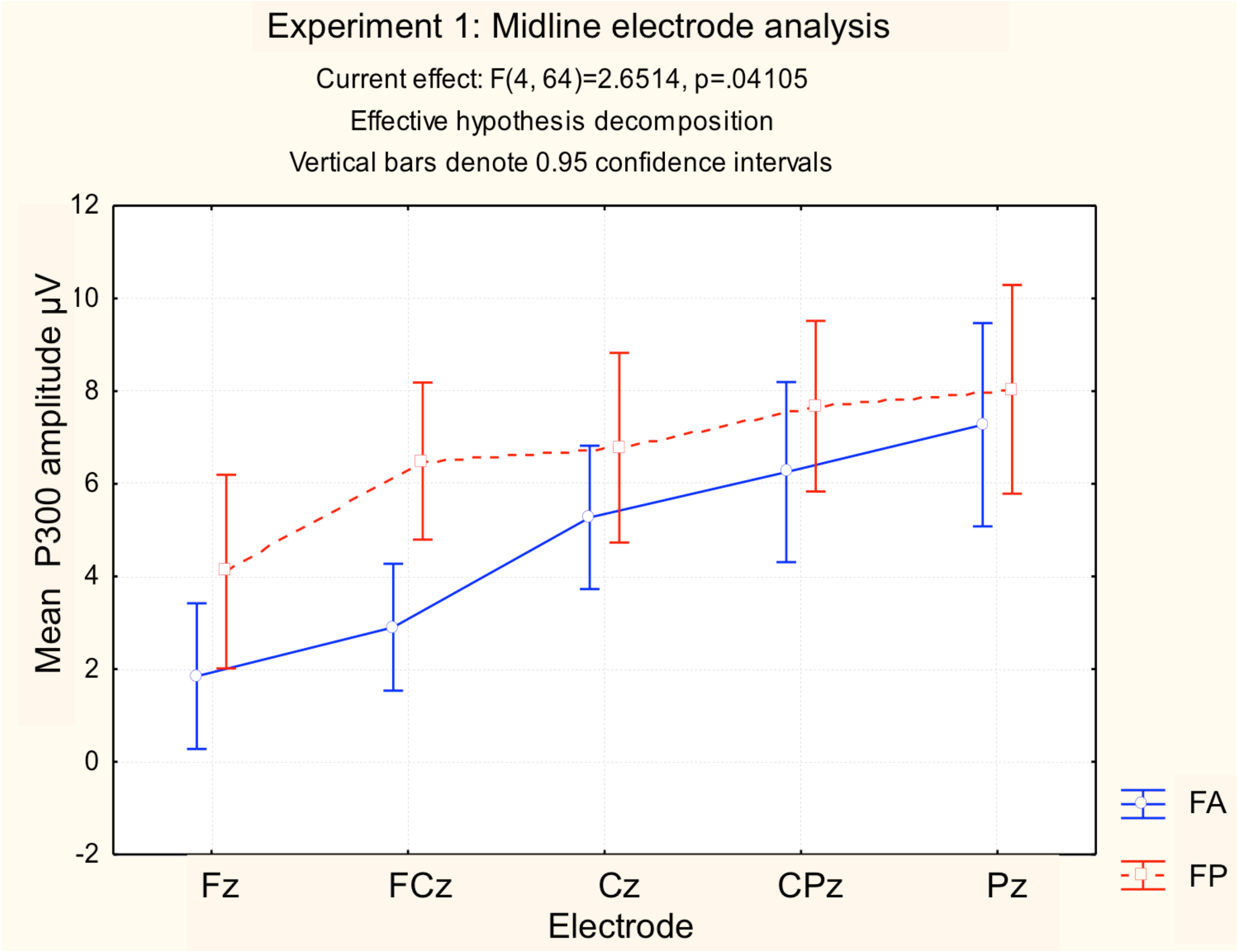
Experiment 1: Midline electrode analysis
Current effect: F(4, 64)=2.6514, p=.04105
Effective hypothesis decomposition
Vertical bars denote 0.95 confidence intervals
Mean P300 amplitude µV
12
10
8
6
4
2
0
-2
Fz
FCz
Cz
CPz
Pz
Electrode
FA
FP

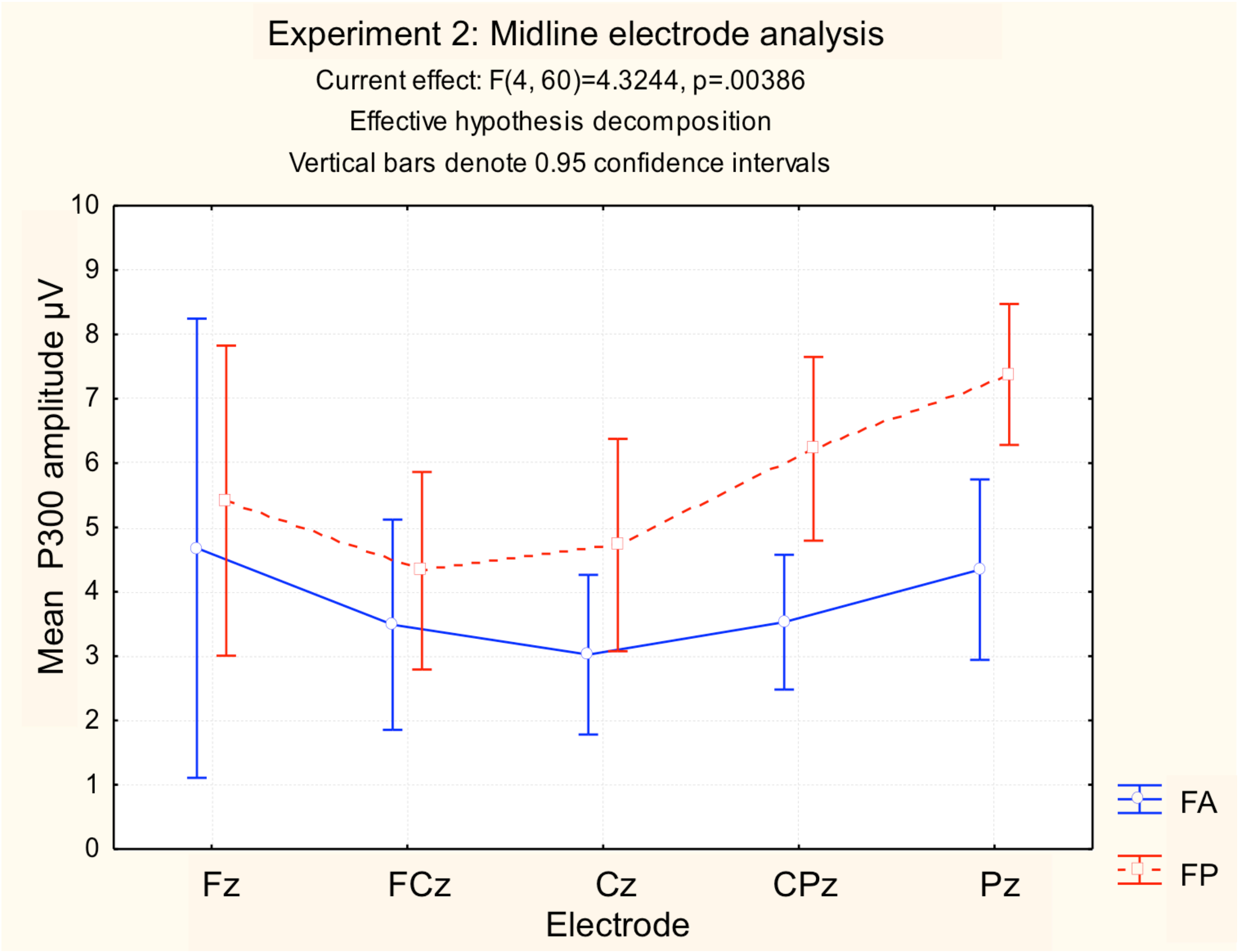
Experiment 2: Midline electrode analysis
Current effect: F(4, 60)=4.3244, p=.00386
Effective hypothesis decomposition
Vertical bars denote 0.95 confidence intervals
Mean P300 amplitude µV
10
9
8
7
6
5
4
3
2
1
0
Fz
FCz
Cz
CPz
Pz
Electrode
FA
FP

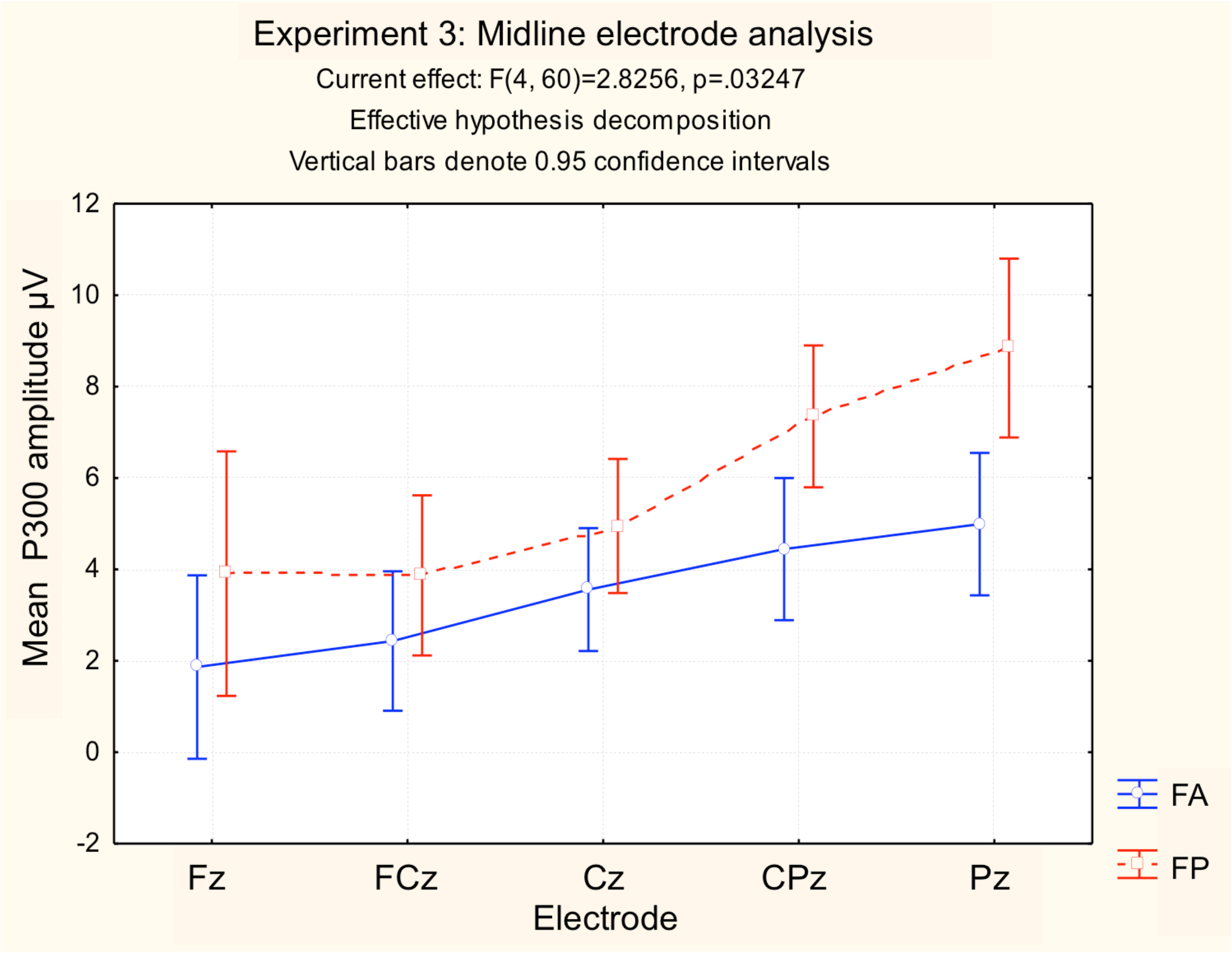

Experiment 3: Midline electrode analysis
Current effect: F(4, 60)=2.8256, p=.03247
Effective hypothesis decomposition
Vertical bars denote 0.95 confidence intervals
Mean P300 amplitude µV
12
10
8
6
4
2
0
-2
Fz
FCz
Cz
CPz
Pz
Electrode
FA
FP

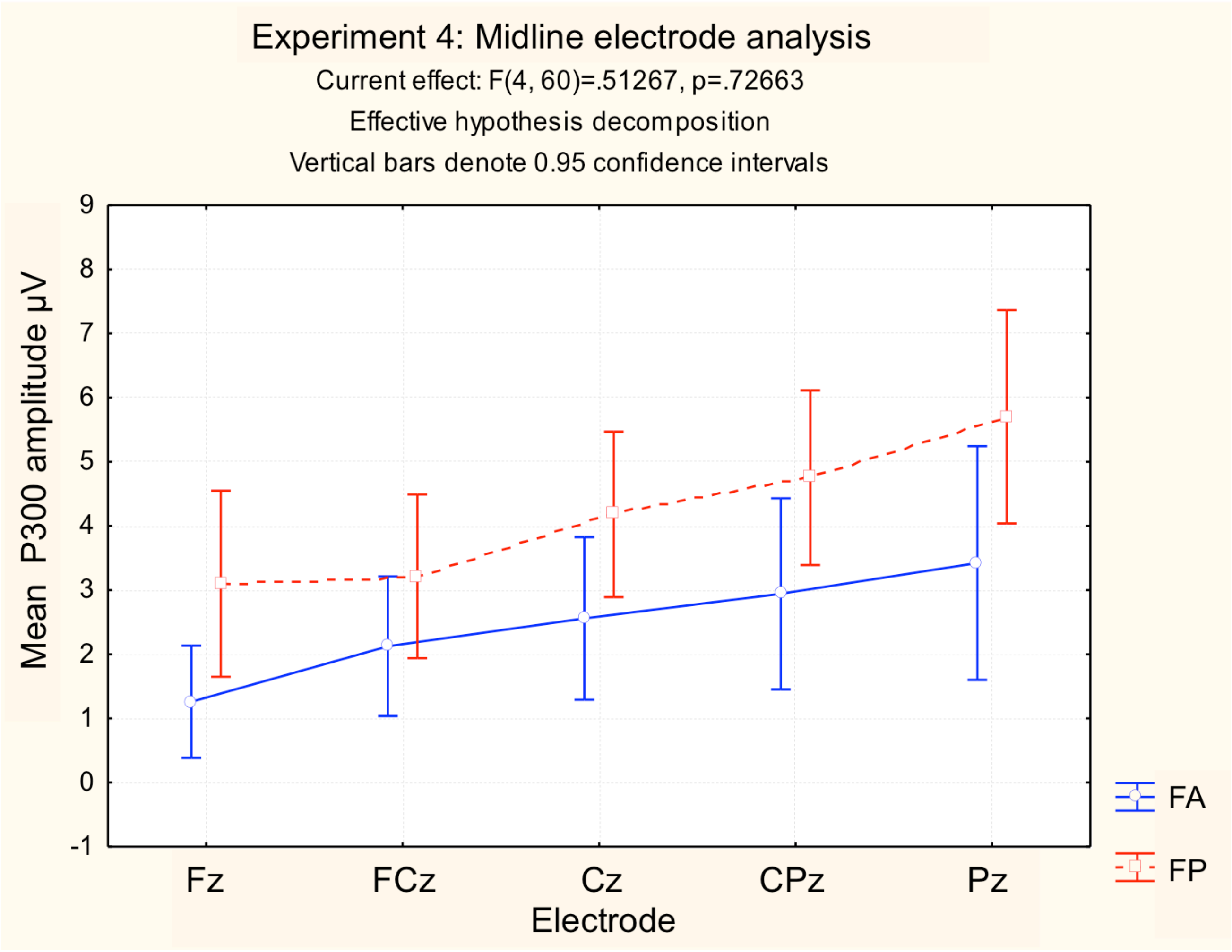
Experiment 4: Midline electrode analysis
Current effect: F(4, 60)=.51267, p=.72663
Effective hypothesis decomposition
Vertical bars denote 0.95 confidence intervals
Mean P300 amplitude µV
9
8
7
6
5
4
3
2
1
0
-1
Fz
FCz
Cz
CPz
Pz
Electrode
FA
FP

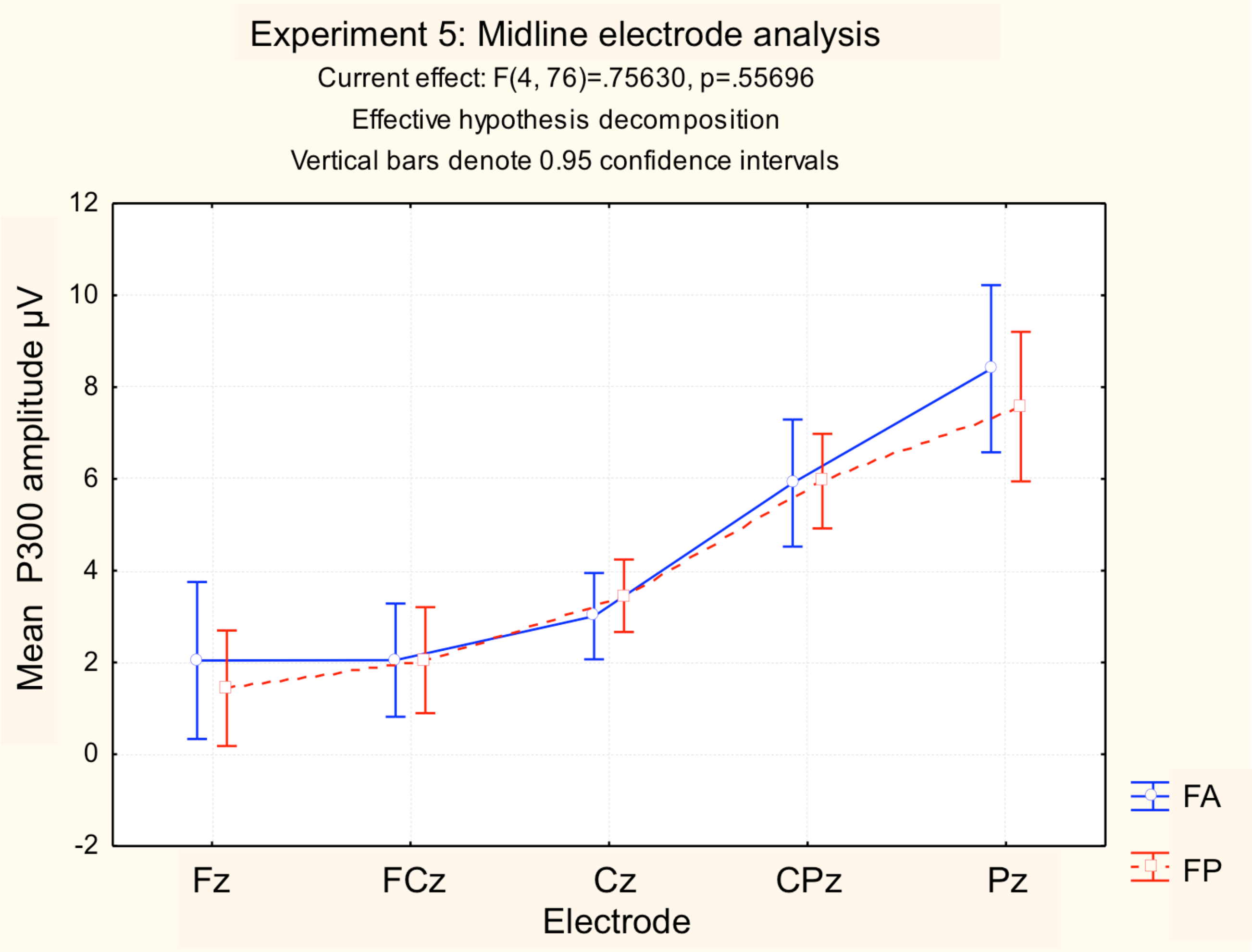

S6 Fig. Interactions of feature present/absent condition with midline electrode location for P300 amplitudes for indicated experiment. FP (blue) indicates feature-present condition; FA (red) indicates feature-absent condition.
